# Iranian general populations' and health care providers' preferences for benefits and harms of statin therapy for primary prevention of cardiovascular disease

**DOI:** 10.1186/s12911-020-01304-w

**Published:** 2020-11-04

**Authors:** Hassan Saadati, Hamid Reza Baradaran, Goodarz Danaei, Afshin Ostovar, Farzad Hadaegh, Leila Janani, Ewout W. Steyerberg, Davood Khalili

**Affiliations:** 1 Department of Epidemiology, School of Public Health, Iran University of Medical Sciences, Tehran, Iran; 2 Ageing Clinical and Experimental Research Team, Institute of Applied Health Sciences, School of Medicine, Medical Sciences and Nutrition University of Aberdeen, Aberdeen, UK; 3 Endocrine Research Center, Institute of Endocrinology and Metabolism, Iran University of Medical Sciences, Tehran, Iran; 4 Department of Global Health and Population and Department of Epidemiology, Harvard TH Chan School of Public Health, Boston, MA USA; 5 Osteoporosis Research Center, Endocrinology and Metabolism Clinical Sciences Institute, Tehran University of Medical Sciences, Tehran, Iran; 6 Prevention of Metabolic Disorders Research Center, Research Institute for Endocrine Sciences, Shahid Beheshti University of Medical Sciences, Tehran, Iran; 7 Department of Biostatistics, School of Public Health, Iran University of Medical Sciences, Tehran, Iran; 8 Department of Biomedical Data Sciences, Leiden University Medical Center, Leiden, The Netherlands; 9 Department of Public Health, Erasmus MC, Rotterdam, The Netherlands; 10 Department of Biostatistics and Epidemiology, Research Institute for Endocrine Sciences, Shahid Beheshti University of Medical Sciences, Tehran, Iran

**Keywords:** Preferences, Statins, Benefit harm outcomes, Primary prevention, Cardiovascular disease

## Abstract

**Background:**

The use of statins for primary prevention of cardiovascular diseases is associated with different benefit and harm outcomes. The aime of this study is how important these outcomes are for people and what people's preferences are.

**Methods:**

We conducted a preference-eliciting survey incorporating a best–worst scaling (BWS) instrument in Iran from June to November 2019. The relative importance of 13 statins-related outcomes was assessed on a sample of 1085 participants, including 913 general population (486 women) and 172 healthcare providers from the population covered by urban and rural primary health care centers. The participants made trade-off decisions and selected the most and least worrisome outcomes concurrently from 13 choice sets; each contains four outcomes generated using the balanced incomplete block design.

**Results:**

According to the mean (SD) BWS scores, which can be (+ 4) in maximum and (− 4) in minimum, in the general population, the most worrisome outcomes were severe stroke (3.37 (0.8)), severe myocardial infarction (2.71(0.7)), and cancer (2.69 (1.33)). While myopathy (− 3. 03 (1.03)), nausea/headache (− 2.69 (0.94)), and treatment discontinuation due to side effects (− 2.24 (1.14)) were the least worrisome outcomes. Preferences were similar between rural and urban areas and among health care providers and the general population with overlapping uncertainty intervals.

**Conclusion:**

The rank of health outcomes may be similar in various socio-cultural contexts. The preferences for benefits and harms of statin therapy are essential to assess benefit-harm balance when recommending statins for primary prevention of cardiovascular diseases.

## Background

Cardiovascular diseases contribute to over 30% of deaths worldwide and more than 40% in Iran [1, 2]. Given that the raised total cholesterol and low-density lipoprotein (LDL) cholesterol are the major risk factors for cardiovascular disease (CVD), controlling hypercholesterolemia is an important target in managing CVD risk [3]. Statins are a class of lipid-lowering drugs that have been widely used for reducing the level of plasma LDL cholesterol [4]. Statins are widely prescribed for the primary and secondary prevention of CVD [5, 6], and their use has been significantly increased over the last 30 years [7].

The various harm and benefit outcomes that are associated with using statins have been reported in previous studies [8–10]. Shared decision-making is a process in which patients and physicians work together to make the best decision for health care, especially when treatments have different benefit and harm outcomes [11]. Statin therapy is a crux for primary prevention of CVD, and its prescription is a preference-sensitive decision [12]. Guidelines provide tools for risk calculation and decision thresholds for preventive drug therapy and recommend shared decision for borderline CVD risks [13, 14]. People value the benefit and harm outcomes of statins differently. This study aimed to elicit the preferences for these benefits and harms in different settings, including rural and urban areas and among healthcare providers and the general population. We used the best–worst scaling technique to elicit preferences; it is a popular method in health for its cognitive and administrative simplicity [15].

## Methods

We conducted a preference-eliciting survey incorporating a best–worst scaling (BWS) instrument in Iran from June to November 2019. The best–worst scaling method was devised by Finn and Louviere [16] and introduced to healthcare research by McIntosh and Louviere [17]. In this method, choice sets are constructed by combining various items and then asking respondents to select the best and worst items from each choice set. According to the choice sets format, BWS is divided into three types: the object case, the profile case, and the multi-profile case [18, 19]. Because in the present study, our purpose was to measure the individual preferences, we used object case, which is suitable for perceiving the relative evaluation of the multiple items the respondents chose.

### Questionnaire design and procedure

In the object case BWS—or maximum difference scaling (MaxDiff) [16], measuring a set of items on an underlying, latent, subjective scale is interested. Thus, this case requires a list of items to be measured. In the current study, we used a list of items consist of 13 statin-associated benefit/harm outcomes including moderate MI, severe MI, moderate stroke, severe stroke, unstable angina, heart failure, liver injury, myopathy, type 2 diabetes, acute kidney failure, cancer, nausea/headache, and treatment discontinuation due to side-effects. These outcomes have been previously selected from RCTs/meta-analysis by Yebyo et al. [8] To equally familiarize respondents with each of the 13 outcomes, we prepared specific clear definitions and short lay descriptions for each outcome. To assess whether the short lay descriptions were clear enough for the individuals, we first consulted experts in the field to check for their appropriateness. Second, we piloted the lay descriptions with 20 individuals aged 40–60 years that helped rephrase and simplify the medical terms; only some minor changes were made in the wording and no substantial revisions were required.

The object case BWS method consists of a series of choice sets, from the list of items, where respondents are asked to select the "best" (or most important) and "worst" (or least important) items in each choice set. In the present study, the terms "best" and "worst" refer to the most and least worrisome clinical outcome, respectively, and respondents are asked to indicate the most and the least worrisome outcome in each choice set. A balanced incomplete block design was used to construct the choice sets so that each possible choice was seen the same number of times through all choice sets, and each choice set included the same number of items [19]. Therefore, each choice has the same possibility to be chosen as the most or least worrisome outcome. This design generated 13 choice sets with four outcomes in each choice set so that each outcome coexisted with another one just once (Appendix). An example of BWS questions is given in the appendix. A preliminary test with 10 participants was conducted to assay the feasibility of the survey design.

### Participants and data collection

Participants were selected from the population covered by primary health care centers in Iran from June to November 2019. We decided IraPEN piloted cities located in four provinces: Naghadeh in West Azerbaijan, Maragheh in East Azerbaijan, Shahreza in Isfahan, and Baft in Kerman Province. IraPEN is an Iranian modified version of the WHO Package of Essential Noncommunicable Diseases Interventions (WHO PEN). It is a part of the national health transformation plan, launched in 2014 by the Ministry of Health to provide universal health coverage, including access to CVD prevention and care [20]. In each city, four urban and four rural health centers were randomly selected. In Maragheh, the number of centers chosen was twice that of the other three cities because the population covered by primary health care centers was almost twice that of the other areas. We planned to recruit 1000 (500 in rural health centers) care-receivers attending to these health centers.

We recruited the participants using the household health files from health centers with a random sampling plan. Participants recruited were 40 years or older without a history of CVD events. The study participants were interviewed face to face in health centers. First, to familiarize participants with the 13 outcomes, simple descriptions were presented and asked respondents to express their perceived severity using a visual analog scale (VAS) for each of the 13 outcomes. We then provided the choice sets designed using the BWS method. In each BWS choice set, the respondent was asked to select the most and least worrisome outcome. We asked all staff at selected health centers to answer the same questionnaire as well. A total of 172 health care providers from urban and rural health centers completed the BWS questionnaire. All participants signed an informed consent form and ethical approval for this study was obtained from the Ethics Committee of the Iran University of Medical Sciences.

### Statistical analysis

We followed the standard analysis of the BWS designs and used a counting approach, followed by statistical modeling, to analyze data [21]. The counting approach calculates several types of scores based on the number of times each item was chosen as "best" and the number of times chosen as "worst" across participants. To analyze item importance, we used the average of "Best minus Worst (B-W)".

The modeling approach, known as the Maximum-difference model (logit model), is an expansion of the conditional logit model. This model assumes that respondents evaluate all possible pairs of items and choose the pair that reflects the maximum difference in preference or importance. Under these assumptions, the probability of selecting the item "i" as the "best" and item "j" as the "worst" expressed as a conditional logit model. In our study, each choice set contained four of 13 outcomes. Therefore, the number of possible pairs in each choice set was 12 pairs. The demographic and clinical characteristics of respondents were reported using descriptive statistics. Also, linear regression was used to evaluate factors, among these characteristics, that affected the preferences. We used R 3.2.2 and STATA 14.0 for data analyses.

## Results

### Characteristics of respondents

We invited 1000 care-receivers from 20 urban and 20 rural health centers. The non-response rate was less than 5%, those with a history of CVD were excluded, and finally, 913 subjects (449 from urban and 464 from rural) were eligible and included in the study. All 172 invited caregivers participated in the study. The socio-demographic and other characteristics of participants are summarized in Table 1. The mean (SD) age of the total participants was 49.8 (8.1) years and 53.2% (n = 486) were female. In terms of education, 97% of the general population participants were literate and 50% educated in middle school or higher (60% in urban and 35% in rural areas). Among the general population, 19.6% reported that they used statins at the time of study or in the past. The mean (SD) age of 172 health care providers was 35.4 (7.5) years, and 82.5% (n = 142) were female. The health care providers' demographic and clinical characteristics were different from those of the general population.

**Table 1:** Characteristics of participants involved in the preference eliciting study

Characteristics	Health care provider n = 172 N (%)	Total population n = 913 N (%)	Urban population n = 449 N (%)	Rural population n = 464 N (%)
Sex male	30 (17.44)	427 (46.77)	201 (44.77)	226 (48.71)
Female	142 (82.56)	486 (53.23)	248 (55.23)	238 (51.29)
Age mean (SD)	35.37 (7.58)	49.78 (8.148)	50.26 (8.28)	49.31 (7.99)
40–59	172 (100.00)	783 (85.76)	381 (84.86)	402 (86.64)
= > 60	0 (0)	130 (14.24)	68 (15.14)	62 (13.36)
*Education*				
Non	0 (0)	29 (3.18)	11 (2.45)	18 (3.88)
Primary	10 (5.81)	451 (49.40)	168 (37.42)	283 (60.99)
Middle school	34 (19.77)	272 (29.79)	150 (33.41)	122 (26.29)
High school and above	128 (74.42)	161 (17.63)	120 (26.73)	41 (8.84)
*Job*				
Salaried	159 (92.44)	121 (13.25)	87 (19.38)	34 (7.33)
Run own business	0 (0)	250 (27.38)	99 (22.05)	151 (32.54)
Pensioned	0 (0)	107 (11.72)	67 (14.92)	40 (8.62)
No job	13 (7.56)	435 (47.56)	196 (43.56)	239 (51.51)
*Co-living person*				
Alone	7 (4.07)	18 (1.97)	8 (1.78)	10 (2.16)
Family	165 (95.93)	895 (98.03)	441 (98.22)	454 (97.85)
*Morbidity*^*a*^				
None	158 (91.86)	631 (69.11)	300 (66.82)	331 (71.34)
Yes	14 (8.14)	282 (30.89)	149 (33.18)	133 (28.66)
*Statin use*				
no	164 (95.35)	739 (80.94)	356 (79.29)	383 (82.54)
Yes now	4 (2.33)	123 (13.47)	62 (13.81)	61 (13.15)
Yes past	4 (2.33)	51 (5.59)	31 (6.90)	20 (4.31)
Marital status single	39 (22.67)	28 (3.07)	8 (1.78)	20 (4.31)
Married	127 (73.84)	842 (92.22)	425 (94.65)	417 (89.87)
Divorced	5 (2.91)	12 (1.31)	6 (1.34)	6 (1.29)
Widow	1 (0.58)	31 (3.40)	10 (2.23)	21 (4.53)
*Family history of heart disease*				
Yes	41 (23.84)	254 (27.82)	138 (30.73)	116 (25.00)
No	131 (76.16)	654 (71.63)	309 (68.82)	345 (74.35)
Don’t know	0 (0)	5 (0.55)	2 (0.45)	3 (0.65)
*Family history of stroke*				
Yes	11 (6.40)	125 (13.69)	60 (13.36)	65 (14.01)
No	161 (93.60)	784 (85.87)	388 (86.41)	396 (85.34)
Don’t know	0 (0)	4 (0.44)	1 (0.22)	3 (0.65)
*Family history of hypertension*				
Yes	104 (60.47)	518 (56.74)	231 (51.45)	287 (61.85)
No	68 (39.53)	390 (42.72)	215 (47.88)	175 (37.72)
Don’t know	0 (0)	5 (0.55)	3 (0.67)	2 (0.43)
*Family history of Hyperlipidemia*				
Yes	76 (44.19)	291 (31.87)	129 (28.73)	162 (34.91)
No	96 (55.81)	612 (67.03)	316 (70.38)	296 (63.79)
Don’t know	0 (0)	10 (1.10)	4 (0.89)	6 (1.29)
*Family history of diabetes*				
Yes	50 (29.07)	295 (32.31)	152 (33.85)	143 (30.82)
No	122 (70.93)	616 (67.47)	296 (65.92)	320 (68.97)
Don’t know	0 (0)	2 (0.22)	1 (0.22)	1 (0.22)

^a^Hypertension, Hyperlipidemia, Type 2 diabetes, and Cancer

### Best- worst scaling survey results

The counting analysis results for each group (health care provider, total population, and subpopulations) are presented in Table 2 as total best, total worst, and best minus worst (B–W) score for each outcome. According to the B–W scores, the most worrisome outcomes were severe stroke, sever MI, and cancer, and the least worrisome outcomes were myopathy, nausea/headache, and treatment discontinuation.

**Table 2:** Importance of 13 outcomes ranked by average B-W scores

Outcomes	Health care providers = 172					Total population = 913				
	Total best	Total worst	B-W score	Mean B-W score	Rank	Total best	Total worst	B-W score	Mean B-W score	Rank
Severe stroke	594	0	594	3.45	1	3119	39	3080	3.37	1
Severe MI	467	1	466	2.71	2	2510	29	2481	2.71	2
Cancer	485	24	461	2.68	3	2868	110	2458	2.69	3
Moderate stroke	212	27	185	1.07	4	1040	134	906	0.99	4
Moderate MI	114	61	53	0.31	5	593	289	304	0.33	5
Type 2 diabetes	45	111	− 66	− 0.38	9	326	498	− 172	− 0.19	6
Liver injury	92	85	7	0.04	6	374	594	− 220	− 0.24	7
Acute kidney failure	99	126	− 27	− 0.16	7	496	725	− 229	− 0.25	8
Heart failure	101	142	− 41	− 0.24	8	589	836	− 247	− 0.27	9
Unstable angina	16	218	− 202	− 1.17	10	152	1238	− 1086	− 1.19	10
Treatment discontinuation	4	422	− 418	− 2.43	11	36	2083	− 2047	− 2.24	11
Nausea/headache	3	459	− 456	− 2.65	12	25	2483	− 2458	− 2.69	12
Myopathy	4	560	− 556	− 3.23	13	41	2811	− 2770	− 3.03	13

Outcomes	Urban population = 449					Rural population = 464				
	Total best	Total worst	B-W score	Mean B-W score	Rank	Total best	Total worst	B-W score	Mean B-W score	Rank
Severe stroke	1526	17	1509	3.36	1	1593	22	1571	3.38	1
Severe MI	1198	16	1182	2.63	3	1312	13	1299	2.80	2
Cancer	1301	38	1263	2.81	2	1267	72	1195	2.57	3
Moderate stroke	540	53	487	1.08	4	500	81	419	0.90	4
Moderate MI	300	119	181	0.40	5	293	170	123	0.26	5
Type 2 diabetes	147	270	− 123	− 0.27	8	179	228	− 49	− 0.11	6
Liver injury	204	278	− 74	− 0.16	6	170	316	− 146	− 0.31	9
Acute kidney failure	250	350	− 100	− 0.22	7	246	375	− 129	− 0.28	8
Heart failure	275	419	− 144	− 0.32	9	314	417	− 103	− 0.22	7
Unstable angina	71	610	− 539	− 1.20	10	81	628	− 547	− 1.18	10
Treatment discontinuation	8	1005	− 997	− 2.22	11	28	1078	− 1050	− 2.26	11
Nausea/headache	9	1271	− 1262	− 2.81	12	16	1212	− 1196	− 2.58	12
Myopathy	8	1391	− 1383	− 3.08	13	33	1420	− 1387	− 2.99	13

The results are based on the number of times each item was chosen as "best" and the number of times chosen as "worst" across participants. We generated 13 choice sets with four outcomes in each choice set so that each outcome coexisted with another one just once. To analyze item importance, we used the average of "Best minus Worst (B-W)"

**Fig. 1:**
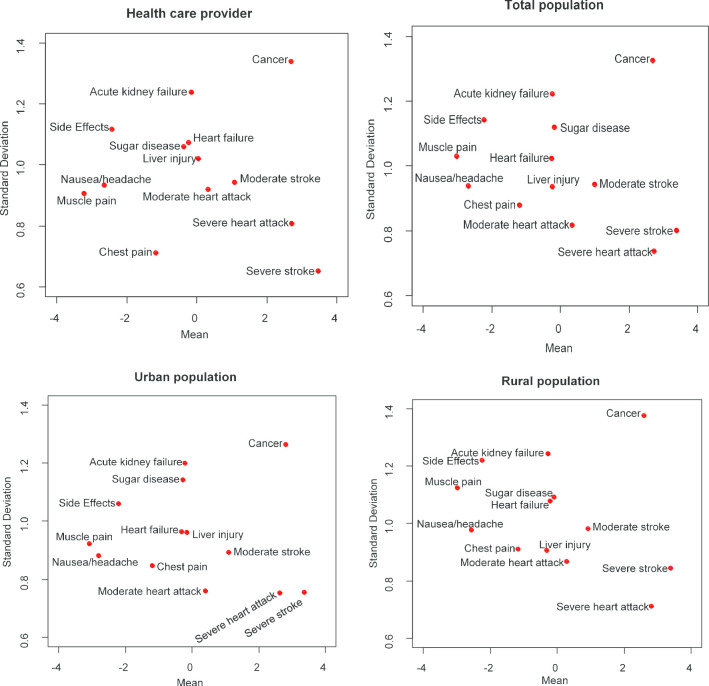
Relationship between the mean Best–Worst score and its standard deviation. The figure depicts the relative importance of the outcomes among individuals according to the B–W scores' means and standard deviations. The higher the mean and lower the variance, the higher and more stable the importance ranking

Figure 1 shows more information regarding the relative importance of outcomes among individuals. Based on the means of the B-W scores of outcomes as well as their standard deviations, severe MI and cancer are similarly important, but the variance of cancer is more remarkable than severe MI; therefore, the importance of cancer differs largely among individuals. Moreover, severe stroke has a relatively high mean with a relatively low standard deviation of the score, which means that the majority mark severe stroke as the most problematic outcome. Bar plots were drawn from B-W scores to show the heterogeneity in more detail (Additional file 1: Figure 2).

**Fig. 2:**
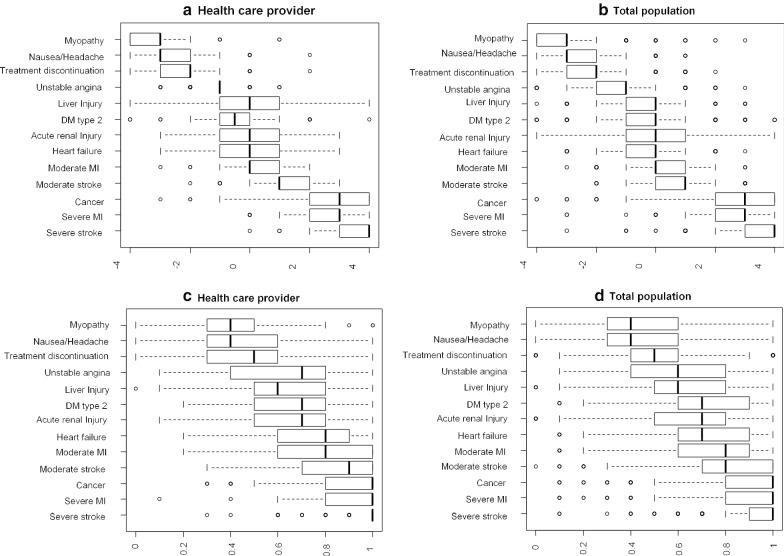
Individual best worst scores (BWS) (**a**, **b**) and Visual Analog Scales (VAS) (**c**, **d**) for the clinical outcomes. The ranking of the 13 outcomes by the median of individual BWS was similar to that of VAS. However, as the box plots show, there is less overlap in the BWS distributions than the VAS ones and the inconsistency of the scores based on the VAS is broader than that based on the BWS

Estimates of statin outcome importance, using the VAS score (bounded by 0 and 1), are presented in Additional file 1: Table 3. Box plots of the BWS scores (on a scale from -4 to 4) and the VAS scores (on a scale from 0–1) are shown in Fig. 2. The ranking of the 13 outcomes by the median of individual BWS was similar to that of VAS. However, as the figure depicts, there is less overlap in the BWS distributions than the VAS ones and the inconsistency of the scores based on the VAS is broader than that based on the BWS.

**Table 3:** Estimation results of Maximum-difference model. ^a^

	Health care provider n = 26,832	Total population n = 142,428	Urban population n = 70,044	Rural population n = 72,384
	Coefficient (95% CI)	Coefficient (95% CI)	Coefficient (95% CI)	Coefficient (95% CI)
Severe stroke	7.85 (7.45–8.23)	6.81 (6.66–6.96)	7.13 (6.90–7.35)	6.58 (6.37–6.78)
Severe MI	6.73 (6.37–7.08)	5.86 (5.72–5.99)	6.06 (5.86–6.26)	5.73 (5.54–5.92)
Cancer	6.62 (6.26–6.97)	5.76 (5.63–5.90)	6.28 (6.07–6.48)	5.34 (5.16–5.52)
Moderate stroke	5.41 (5.08–5.75)	4.52 (4.39–4.65)	4.91 (4.72–5.10)	4.20 (4.03–4.37)
Moderate MI	4.46 (4.13–4.78)	3.69 (3.57–3.82)	4.05 (3.86–4.23)	3.41 (3.25–3.57)
Heart failure	4.11 (3.79–4.42)	3.28 (3.16–3.40)	3.51 (3.33–3.68)	3.11 (2.95–3.26)
Acute kidney failure	4.16 (3.84–4.48)	3.27 (3.15–3.39)	3.58 (3.40–3.76)	3.02 (2.86–3.18)
Type 2 diabetes	3.60 (3.28–3.92)	3.18 (3.06–3.30)	3.32 (3.13–3.50)	3.08 (2.92–3.25)
Liver injury	4.04 (3.72–4.36)	2.93 (2.81–3.05)	3.26 (3.08–3.44)	2.67 (2.51–2.82)
Unstable angina	2.42 (2.15–2.70)	1.94 (1.84–2.05)	2.08 (1.90–2.23)	1.84 (1.70–1.98)
Treatment discontinuation	0.90 (0.67–1.13)	0.80 (0.71–0.90)	.90 (0.76–1.04)	.72 (0.59–0.85)
Nausea/headache	0.67 (0.44–0.90)	0.36 (0.27–0.46)	.30 (0.16–0.44)	.42 (0.28–0.55)
Myopathy	Ref	Ref	Ref	Ref

^a^ The coefficients are related to a conditional logit model. The exponential of the coefficients shows the odds ratio of choosing an item as the most worrisome outcome compared to choosing the Myopathy as the most worrisome one across all choice sets

Table 3 shows the relative importance of the 13 outcomes associated with statin use estimated by the Maximum-difference model. The importance of each outcome was assessed relative to the "Myopathy," which was consistently rated as the least worrisome outcome. The results revealed that severe stroke, sever MI, and cancer, which are the most worrisome outcomes of the 13 outcomes associated with statin use in the general population, are approximately 905, 349, and 318 times as worrisome as myopathy, respectively (these numbers are exponential of the coefficients in Table 3).

Table 4 presents data on self-reported difficulties in understanding and compilation of the BWS questionnaire. Most of the participants rated the BWS task as easy-to-understand and easy-to-answer. They also reported that answers to all questions were, in a way, consistent with their preferences. Participants stated that the reasons that influence their choices were "Availability of medical care", "Severity of the clinical outcome", "Curability of the clinical outcome", "Long-term consequences of the clinical outcome", and "Cost-effectiveness of medical services" in order. Also, in the multiple linear regression model used to evaluate associations between respondent characteristics and choosing an outcome as the most worrisome, we did not find any significant association between the factors and the outcome preferred (Additional file 1: Table 4).

**Table 4:** Rating of the best–worst scaling exercise

Variable	Items	Health care provider		Total population	
		N = 172	%	N = 913	%
Questions were easy to understand	Strongly agree	92	53.49	248	27.16
	Agree	60	34.88	247	52.35
	Neither agree nor disagree	9	5.23	147	16.10
	Disagree	8	4.65	36	3.94
	Strongly disagree	3	1.47	4	0.44
Questions were easy to answer	Strongly agree	78	45.35	253	27.71
	Agree	70	40.70	503	55.09
	Neither agree nor disagree	12	6.98	123	13.47
	Disagree	9	5.23	28	3.07
	Strongly disagree	3	1.74	6	0.66
Answers were consistent with my preferences	Strongly agree	100	58.14	384	42.06
	Agree	64	37.21	486	53.23
	Neither agree nor disagree	5	2.91	40	4.38
	Disagree	3	1.74	3	0.33
	Strongly disagree	0	0	0	0
Main reasons for influencing preference for health problems	Availability of medical care	41	23.84	285	31.22
	Severity of health problems	45	26.16	223	24.42
	Curability	34	19.77	134	14.68
	Long-term consequences	33	19.19	117	12.81
	Cost-effectiveness of medical services	19	11.05	154	16.87

This table presents data on self-reported difficulties in understanding and compilation of the BWS questionnaire regarding different aspects

## Discussions

Hypercholesterolemia is a major and modifiable risk factor for CVD, and medication to lower LDL cholesterol is a crucial recommendation for preventing CVD in many guidelines. We studied the importance of different health outcomes related to statin therapy from the health care providers' and the general population's perspective in urban and rural areas. Each outcome's quantitative weight was derived, showing that the outcomes are scored and ranked similarly in various socio-cultural contexts.

Statin therapy is the first line of treatment for the primary prevention of CVD based on the absolute risk [22, 23]. However, the threshold of absolute risk of CVD over ten years has lowered to 7.5–10% for this medication [13, 24]. This decrease in the threshold has increased the number of people eligible for statin therapy that most of them may be healthy and statins may not be appropriate for them. Although using statins in individuals at risk can prevent cardiovascular disease, there are risks of different harm outcomes like muscle pain, increasing serum blood sugar, etc. [8]. Assessing relative importance for treatment outcomes is the key step in doing a benefit-harm assessment, informed by patient preferences [25].

Patients value different health outcomes at different importance. An individual's preference reflects the degree of his/her subjective satisfaction, distress, or desirability for a given health outcome [26]. The trade-offs between different benefits and harms of treatments are thus largely influenced by how patients place the relative importance on each outcome [27].

We found that people without a history of CVD events considered some statin-related outcomes more worrying than others. Severe stroke, severe MI, and cancers were ranked as the most worrisome outcomes, while treatment discontinuation, nausea/headache, and myopathy were considered the least. There were essentially no differences in the ranking by health care providers and the general population. Also, we found that respondents' preferences in urban and rural areas were similar, despite large differences in the socio-economic contexts. These results were the same as the study by Yebyo et al. carried out in Ethiopia and Switzerland. [8]. They found similar preferences between Ethiopia's and Switzerland's population with overlapping uncertainty intervals. Although more studies in different countries are recommended to ensure these results' generalizability, it seems that the preferences are consistent across dissimilar settings.

Assessing individuals' preferences provides valuable information in promoting preventive campaigns. There has been growing interest in using preference elicitation methods to inform health policy and medical decision-making in recent years [28, 29]. Our results help distinguish between more and less worrying outcomes from the patients' perspective and inform decision-making on the preventive treatment of individuals with risk for cardiovascular events. In this way, quantitative weights can be used to calculate a net benefit for statin therapy. Yebyo et al. used this kind of weights and found the optimum risk thresholds for statin therapy. For instance, they found that at the risk threshold between 12 and 21%, depending on age and sex, weighted harms and benefits of statin therapy are equal for patients based on their own preferences [30].

Although the main economic evaluation method is cost-utility analysis, including the standard gamble and time trade-off, such an approach is not suitable to find trade-offs between health attributes to evaluate current practices. Ordinal preference elicitation methods, including Discrete Choice Experiments and ranking methods, are therefore commonly used in health economics and health service research [31]. The BWS is relatively simple to understand and reduce the cognitive burden for respondents and facilitates the evaluation of maximum-difference questions [15]. BWS also overcomes the traditional 'pick one' task used in Discrete Choice Experiments by eliciting additional information on both the most and least preferred option [32]. Additionally, BWS possesses the ability to embrace a larger set of factors to determine preferences [33]. However BWS has some limitations such as questions about its theoretical foundations, uncertainty about its ability to predict consumer choice, getting repetitive for some if many objects/attributes, and relatively burdensome to some respondents [34].

Compared to the VAS, the BWS is more suitable for ranking the outcomes' relative importance. One advantage of using BWS to elicit preferences is that it allowed us to ask patients in a way that they can make trade-offs between outcomes [16]. In contrast, there are no trade-offs involved in VAS tasks when doing it and maybe less sensitive to detect differences in the outcomes' scores [29]. In other words, most patients can complete BWS tasks to express their preferences without major problems. Our results also showed that VAS is lesser reliable with broader distribution in comparison to BWS.

The main limitation in using the BWS method is that a different understanding of the outcomes would influence eliciting the preferences in this approach [28]. Therefore, one of the challenges when designing the questionnaire was to ensure that respondents had a common understanding of the study outcomes when they were doing preference-elicitation tasks. For this purpose, we asked respondents to read the short lay descriptions for each outcome carefully and complete the VAS tasks before they did the BWS tasks. Considering participants from health care centers, and not both public and private sections, is a limitation in our study. However, we showed that individual characteristics, including education and job, had no association with their preferences.

## Conclusion

With the preference study results, we know how individuals consider the trade-offs between treatment benefits and harms. We found that our survey respondents consistently ranked severe stroke, severe MI, and cancers as the most worrisome outcomes while treatment discontinuation, nausea/headache, and myopathy as the least worrisome outcomes. The quantitative weights derived here for harms and benefits of statin therapy would be beneficial to determine the eligible people for statin therapy, mainly in a policy perspective. Our results showed that people from different socio-cultural contexts, rank the health outcomes the same way.

## Supplementary information

**Additional file 1**.This contains supplementary tables and figures, including an example BWS questionnaire, balanced incomplete block design of outcomes, detailed results of counting analysis, Box plots of the individual BWS scores, results of linear regression assessing the influence of participants' characteristics on preference values , and the questionair

## Data Availability

The datasets used and/or analysed during the current study are available from the corresponding author on reasonable request.

## Abbreviations

BWS: Best–worst scaling;
LDL: Low-density lipoprotein
CVD: Cardiovascular disease
WHO: World health organization
VAS: Visual analog scale
