# Supplementary material for: Iranian general populations' and health care providers' preferences for benefits and harms of statin therapy for primary prevention of cardiovascular disease
Source: BMC Med Inform Decis Mak. 2020 Nov 4;20:288. doi: 10.1186/s12911-020-01304-w (PMC7640674; doi:10.1186/s12911-020-01304-w)
Supplement: Supplementary file 1 — Additional file 1.This contains supplementary tables and figures, including an example BWS questionnaire, balanced incomplete block design of outcomes, detailed results of counting analysis, Box plots of the individual BWS scores, results of linear regression assessing the influence of participants' characteristics on preference values , and the questionair [file 12911_2020_1304_MOESM1_ESM.docx]

**Iranian general populations' and health care providers' preferences for benefits and harms of statin therapy for primary prevention of cardiovascular disease**

Hassan Saadati^1^, Hamid Reza Baradaran^1,2,3^*, Goodarz Danaei^4^, Afshin Ostovar^5^, Farzad Hadaegh^6^, Leila Janani^7^, Ewout W. Steyerberg^8, 9^, Davood Khalili^6, 10^*

1-Department of Epidemiology, School of Public Health, Iran University of Medical Sciences, Tehran, Iran

2- Ageing Clinical and Experimental Research Team, Institute of Applied Health Sciences, School of Medicine, Medical Sciences and Nutrition University of Aberdeen, Aberdeen, UK

3- Endocrine Research Center, Institute of Endocrinology and Metabolism, Iran University of Medical Sciences, Tehran, Iran

4- Department of Global Health and Population and Department of Epidemiology, Harvard TH Chan School of Public Health, Boston, MA , **USA**

5- Osteoporosis Research Center, Endocrinology and Metabolism Clinical Sciences Institute, Tehran University of Medical Sciences, Tehran, Iran

6- Prevention of Metabolic Disorders Research Center, Research Institute for Endocrine Sciences, Shahid Beheshti University of Medical Sciences, Tehran, Iran

7- Department of Biostatistics, School of Public Health, Iran University of Medical Sciences, Tehran, Iran

8- Department of Biomedical Data Sciences, Leiden University Medical Center, Leiden, the Netherlands

9- Department of Public Health, Erasmus MC, Rotterdam, The Netherlands

10- Department of Biostatistics and Epidemiology, Research Institute for Endocrine Sciences, Shahid Beheshti University of Medical Sciences, Tehran, Iran

^*^**Corresponding Authors:**

**Davood Khalili**

Department of Biostatistics and Epidemiology, Research Institute for Endocrine Sciences, Shahid Beheshti University of Medical Sciences, Tehran, Iran

Tel: +989128148865

Fax: +982122416264

Emails: dkhalili@endocrine.ac.ir ; dkhalili@hsph.harvard.edu

**Hamid Reza Baradaran**

Department of Epidemiology, School of Public Health, Iran University of Medical Sciences, Tehran, Iran.

Tel: +989122979926

Emails: baradaran.hr@iums.ac.ir ; hamid.baradaran@abdn.ac.uk

**Best Worst scaling questionnaire**

We performed a preference-eliciting survey using the object case (case 1) BWS, which is suitable for perceiving the relative evaluation of the multiple items the respondents chose. In this survey, our purpose was to measure the seriousness of the 13 statin-associated benefit harm outcomes. Supp. Table 1 shows the clinical outcomes used in this survey. After listing the items (outcomes) for respondents' evaluation, a number of different subsets of the items were made from the list according to the Balanced Incomplete Block Design (BIBD). As shown in Supp. Table 2, we generated 13 subsets using BIBD. Each of the subsets was presented as a choice set (questions) to the respondents, who were asked to select the best (or most worrisome) item and the worst (or least worrisome) item in the choice set. This question was repeated until all the subsets have been evaluated. An example of one choice set presented in Supp. Fig. 1, from which the participants had to select their best and worst outcome.

Supp. Table 1: Items used in BW scaling

| Items no. | Outcomes |
| --- | --- |
| 1 | Moderate MI |
| 2 | Severe MI |
| 3 | Moderate stroke |
| 4 | Severe stroke |
| 5 | Unstable angina |
| 6 | Heart failure |
| 7 | Liver injury |
| 8 | Myopathy |
| 9 | Treatment discontinuation |
| 10 | Type 2 diabetes |
| 11 | Acute kidney failure |
| 12 | Cancer |
| 13 | Nausea/headache |

Supp. Table 2: Balanced incomplete block designs (BIBDs), 13 choice sets with four items

| Choice set no. | Items no. | | | |
| --- | --- | --- | --- | --- |
| 1 | 3 | 6 | 1 | 12 |
| 2 | 5 | 9 | 7 | 6 |
| 3 | 7 | 8 | 12 | 10 |
| 4 | 11 | 12 | 13 | 9 |
| 5 | 12 | 5 | 4 | 2 |
| 6 | 1 | 11 | 5 | 8 |
| 7 | 9 | 1 | 10 | 4 |
| 8 | 10 | 13 | 3 | 5 |
| 9 | 13 | 7 | 2 | 1 |
| 10 | 6 | 4 | 8 | 13 |
| 11 | 2 | 10 | 6 | 11 |
| 12 | 4 | 3 | 11 | 7 |
| 13 | 8 | 2 | 9 | 3 |

| Among the following problems, which would worry you most and which would worry you least if they were happened to you? | | |
| --- | --- | --- |
| Most worrisome(Choose one) | Health problems | Least worrisome(Choose one) |
| 🞎 | Liver injury | 🞎 |
| 🞎 | Muscle pain | 🞎 |
| 🞎 | Cancer | 🞎 |
| 🞎 | Sugar disease | 🞎 |

Supp. Fig. 1: Example of choice set.

**Results of counting analysis**

To understand the relative importance of outcomes among individuals, we presented the combination of outcome importance and heterogeneity in the main text. Based on the means of the B-W scores of outcomes as well as their standard deviations, sever MI and cancer are similarly important, but the variance of cancer is larger than sever MI. Therefore, the importance of cancer differs largely among individuals. Also, sever stroke has a relatively high mean with a relatively low standard deviation of the score, which means that the majority prefer to sever stroke as the most worrisome outcome. To show the heterogeneity in detail, we drew bar plots from B-W scores (supp. Fig.2).

Supp. Fig. 2: Distribution of simple Best-Worst scores by items.

Rural population

Health provider


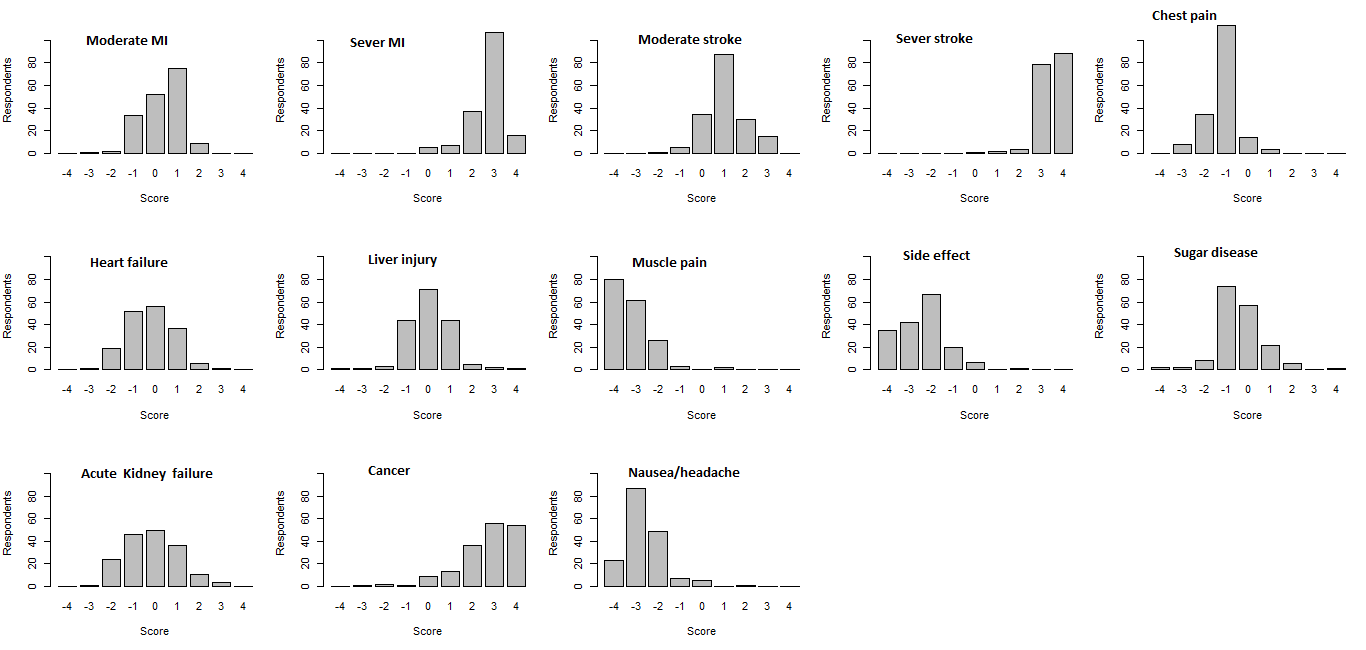


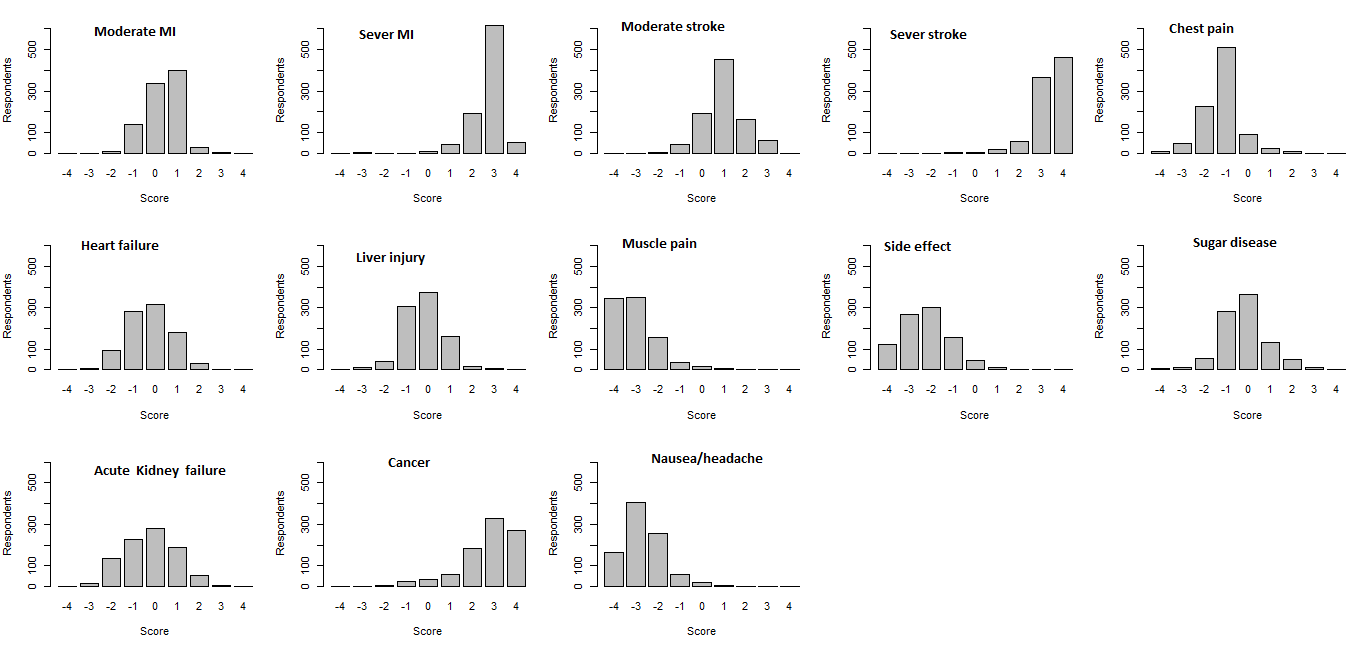


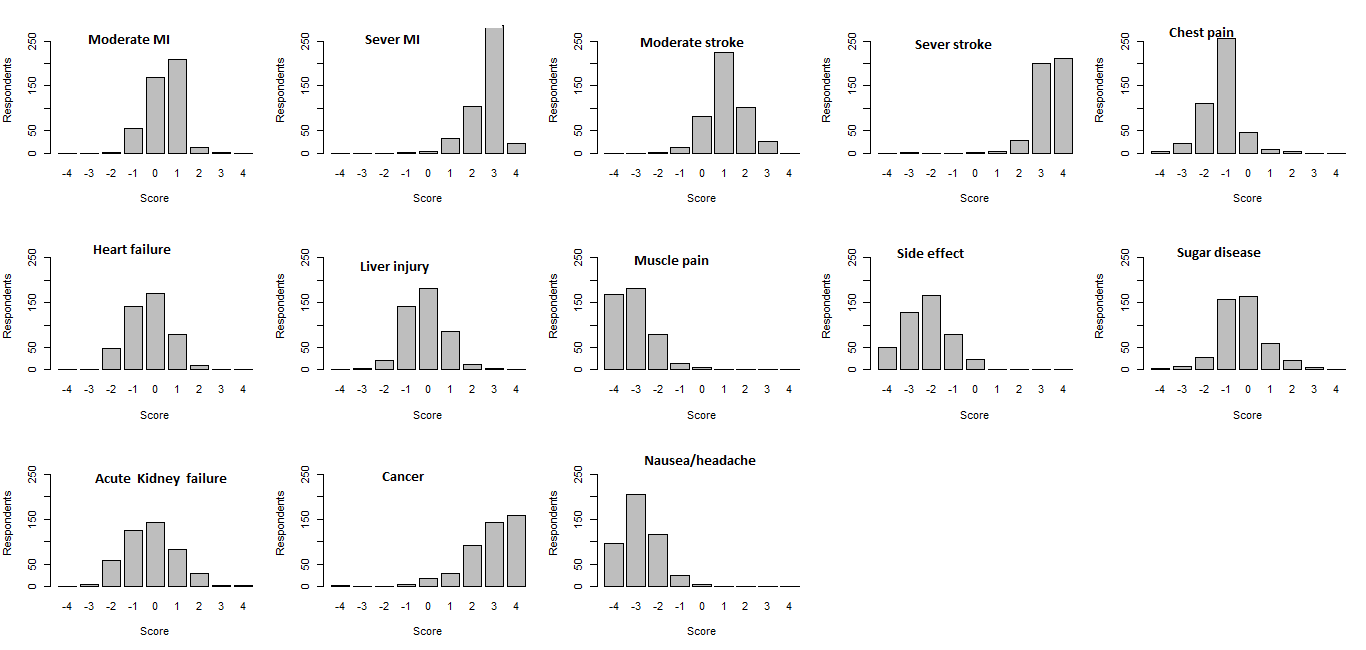


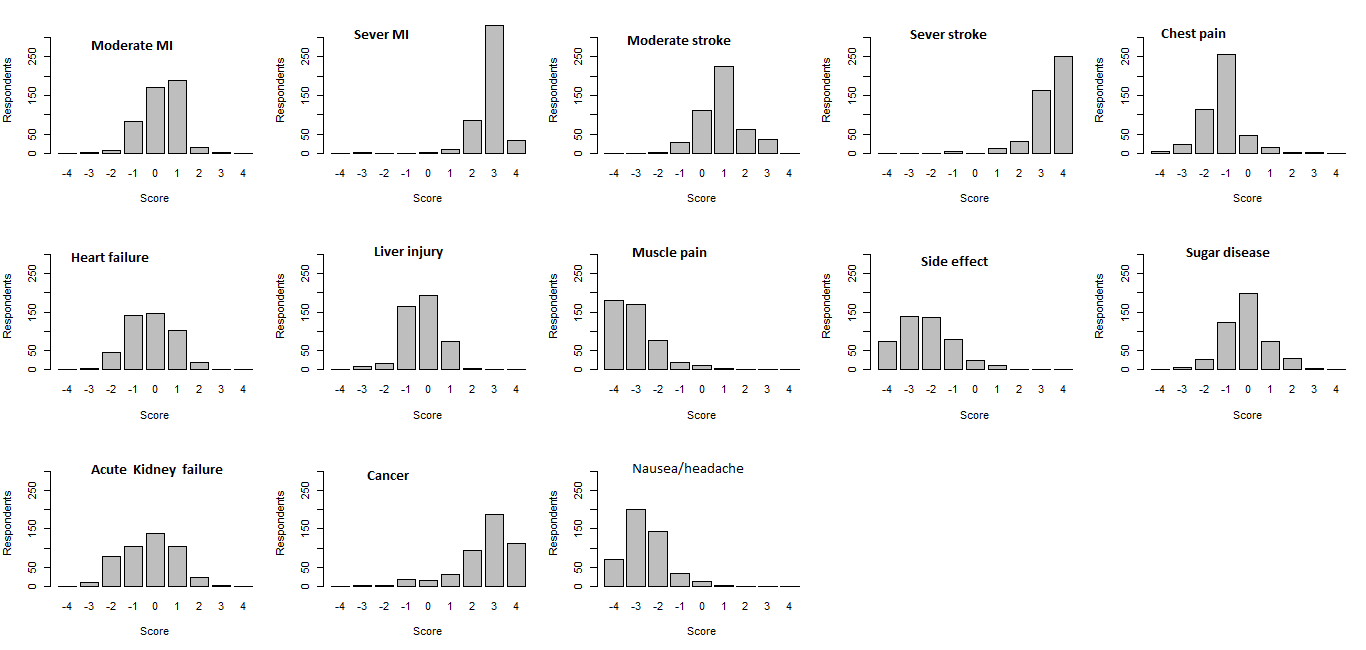


Urban population

Total population

Estimates of statin outcome importance, using VAS score, are presented in Supp. Table 3. The mean, SD and median of the VAS scores (bounded by 0 and 1) are presented.

Supp. Table 3: preference weights based on visual analog scale (VAS)

| Benefit or harm outcomes related to statins | Health provider n= 172 | Total population n=913 | Urban population n= 449 | Rural population n= 464 |
| --- | --- | --- | --- | --- |
| Severe stroke  Mean  SE  Median | .94  .009  1 | .94  .004  1 | .95  .0091  1 | .94  .005  1 |
| Severe MI  Mean  SE  Median | .90  .0113  1 | .89  .005  1 | .91  .007  1 | .88  .007  1 |
| Cancer  Mean  SE  Median | .88  .0113  1 | .89  .005  1 | .90  .008  1 | .88  .007  1 |
| Moderate stroke  Mean  SE  Median | .83  .0131  0.9 | .79  .006  0.8 | .82  .009  0.9 | .76  .009  0.8 |
| Moderate MI  Mean  SE  Median | .75  .0158  0.8 | .73  .007  0.8 | .76  .010  0.8 | .69  .010  0.7 |
| Heart failure  Mean  SE  Median | .75  .014  0.8 | .73  .006  0.7 | .74  .009  0.8 | .71  .008  0.7 |
| Acute kidney failure  Mean  SE  Median | .65  .015  0.7 | .65  .007  0.7 | .66  .010  0.7 | .63  .010  0.6 |
| Type 2 diabetes  Mean  SE  Median | .67  .015  0.7 | .72  .006  0.7 | .75  .009  0.8 | .69  .008  0.7 |
| Liver injury  Mean  SE  Median | .64  .017  0.6 | .63  .007  0.6 | .65  .010  0.7 | .62  .010  0.6 |
| Unstable angina  Mean  SE  Median | .63  .019  0.7 | .60  .008  0.6 | .63  .011  0.6 | .58  .011  0.6 |
| Treatment discontinuation  Mean  SE  Median | .47  .016  0.5 | .49  .007  0.5 | .52  .010  0.5 | .46  .009  0.5 |
| Nausea/headache  Mean  SE  Median | .44  .016  0.4 | .44  .006  0.4 | .48  .009  0.5 | .41  .009  0.4 |
| Myopathy  Mean  SE  Median | .43  .015  0.4 | .45  .006  0.4 | .49  .010  0.5 | .42  .009  0.4 |

**Associations between respondent Characteristics and Preferences Scores:**

Supp. Table 4 shows the results from the multiple linear regression models for all the outcomes. We regressed standardized best minus worst (B-W) scores for each outcome on socio-demographic and other factors using linear regression to evaluate associations. But there was no consistent significant association between the respondent characteristics and the B-W scores.

Supp. Table 4: Association of participants" characteristics with standardized B-W scores of each clinical outcome

| Characteristics | Moderate MI | Severe MI | Moderate stroke | Severe stroke | Unstable angina | Heart failure | Liver injury |
| --- | --- | --- | --- | --- | --- | --- | --- |
| Respondent group |  |  |  |  |  |  |  |
| Population | Ref |  |  |  |  |  |  |
| Health provider | 0.004 (-.0.4,0.05) | 0.015 (-0.03,0.06) | -0.039 (-0.09,0.02) | 0.041 (-0.01,0.09) | -0.009 (-0.06,0.04) | -0.017 (-0.08,0.05) | 0.077 (0.02,0.13)* |
| Sex |  |  |  |  |  |  |  |
| Female | Ref |  |  |  |  |  |  |
| Male | -0.013 (-0.05,0.02) | 0.022 (-0.01,0.06) | 0.002 (-0.04,0.04) | 0.016 (-0.02,0.05) | -0.045 (-0.08,-0.01) * | 0.013 (-0.03,0.06) | 0.002 (-0.04,0.04) |
| Job |  |  |  |  |  |  |  |
| Salaried | Ref |  |  |  |  |  |  |
| Run own business | -0.017 (-0.06,0.03) | 0.007 (-0.03, 0.05) | -0.067 (-0.12,-0.02)* | -0.0002 (-0.04,0.04) | 0.005 (-0.04,0.05) | -0.007 (-0.06,0.05) | -0.017 (-0.07,0.04) |
| Pensioned | -0.014 (-0.07,0.04) | -0.044 (-0.09,0.01) | -0.024 (-0.04,0.04) | -0.023 (-0.08,0.03) | 0.002 (-0.06,0.06) | -0.024 (-0.09,0.05) | 0.054 (-0.01,0.11) |
| No job | -0.022 (-0.06,0.02) | 0.007 (-0.03,0.05) | -0.086 (-0.14,-0.04)* | 0.015 (-0.03,0.06) | -0.023 (-0.07,0.02) | 0.006 (-0.05,0.06) | 0.027 (-0.02,0.07) |
| Comorbidity |  |  |  |  |  |  |  |
| None | Ref |  |  |  |  |  |  |
| Some | 0.008 (-0.02,0.04) | -0.007 (-0.04,0.02) | 0.013 (-0.03,0.05) | 0.004 (-0.03,0.04) | -0.006 (-0.04,0.03) | -0.035 (-0.08,0.01) | -0.028 (-0.07,0.01) |
| Coliving person |  |  |  |  |  |  |  |
| Alone | Ref |  |  |  |  |  |  |
| Family member | 0.003 (-0.08,0.09) | 0.025 (-0.05,0.10) | -0.035 (-0.13,0.06) | 0.025 (-0.05,0.10) | -0.030 (-0.12,0.05) | -0.128 (-0.23,-0.02) | 0.057 (-0.04,0.15) |
| Statin use |  |  |  |  |  |  |  |
| No | Ref |  |  |  |  |  |  |
| Yeas | -0.010 (-0.05,0.03) | 0.001 (-0.04,0.4) | -0.014 (-0.06,0.03) | 0.008 (-0.03,0.04) | 0.008 (-0.03,0.05) | 0.043 (-0.01.0.1) | -0.007 (-0.05,0.04) |
| Age(yrs) | 0.002 (0.001,0.003)* | 0.001 (-0.001,0.002) | -0.0003 (-0.002,0.001) | 0.0007 (-0.03,0.04) | 0.0001 (-0.001,0.002) | -0.001 (-0.003,0.001) | -0.121 (-0.26,0.02) |

Supp. Table 4: cont.

| Characteristics | Myopathy | Treatment discontinuation | Type 2 diabetes | Acute kidney failure | Cancer | Nausea/headache |
| --- | --- | --- | --- | --- | --- | --- |
| Respondent group |  |  |  |  |  |  |
| Population | Ref |  |  |  |  |  |
| Health provider | -0.057 (-0.12,0.004) | -0.012 (-0.08,0.06) | -0.011 (-0.08,0.06) | 0.030 (-0.04,0.10) | -0.041 (-0.12,0.04) | 0.020 (-0.03,0.07) |
| Sex |  |  |  |  |  |  |
| Female | Ref |  |  |  |  |  |
| Male | 0.029 (-0.01,0.07) | 0.005 (-0.04,0.05) | -0.020 (-0.07,0.03) | 0.006 (-0.05,0.06) | -0.054 (-0.11,0.0005) | 0.036 (-0.005,0.08) |
| Job |  |  |  |  |  |  |
| Salaried | Ref |  |  |  |  |  |
| Run own business | -0.052 (-0.12,0.003) | 0.034 (-0.02,0.09) | 0.59 (-0.001,0.12) | -0.018 (-0.08,0.05) | 0.040 (-0.03,0.11) | 0.033 (-0.01,0.08) |
| Pensioned | -0.022 (-0.09,0.04) | 0.011 (-0.06,0.09) | 0.056 (-0.02,0.13) | -0.019 (-0.10,0.06) | 0.042 (-0.05,0.13) | 0.005 (-0.05,0.07) |
| No job | -0.008 (-0.06,0.04) | 0.020 (-0.04,0.08) | 0.090 (0.03,0.14)* | -0.035 (-0.10,0.03) | -0.047 (-0.11,0.02) | 0.054 (0.005,0.10)* |
| Comorbidity |  |  |  |  |  |  |
| None | Ref |  |  |  |  |  |
| Some | 0.013 (-0.02,0.05) | 0.020 (-0.02,0.06) | -0.026 (-0.07,0.02) | -0.010 (-0.06,0.04) | 0.073 (0.02,0.012)* | -0.019 (-0.05,0.02) |
| Coliving person |  |  |  |  |  |  |
| Family member | Ref |  |  |  |  |  |
| Alone | -0.091 (-0.019,0.001) | -0.027 (-0.14,0.08) | 0.030 (-0.08,0.14) | 0.022 (-0.10,0.14) | 0.067 (-0.06,0.20) | 0.080 (-0.01,0.17) |
| Statin use |  |  |  |  |  |  |
| No | Ref |  |  |  |  |  |
| Yeas | -0.005 (-0.05,0.04) | -0.009 (-0.06,0.04) | 0.001 (-0.05,0.05) | 0.030 (-0.03,0.09) | -0.031 (-0.09,0.03) | -0.015 (-0.06,0.02) |
| Age(yrs) | 0.0002 (-0.002,0.002) | 0.001 (-0.001,0.003) | -0.0007 (-0.003,0.001) | 0.001 (-0.001,0.004) | -0.002 (-0.005,0.0005) | -0.001 (-0.003,0.001) |

Ref: Reference group

**Best-Worst Questionnaire for STATINS**

# Eliciting patient preferences on statin related health problems in Switzerland and Ethiopia

This study aims to help us understand your preferences on selected health problems related to use of statins. Statins are class of drugs that lowers your cholesterol level. They are prescribed for individuals to prevent future heart and vascular diseases (heart attack, chest pain, stroke, and heart failure). However, they have also associated adverse effects such as muscle diseases, liver injury, kidney injury, diabetes mellitus and cancers. A need arises to evaluate the balance between the benefits and adverse effects. Considering values and preferences of the different outcomes play important role in deciding whether statins are more of beneficial or harmful. Thus in this study we need to understand the preferences of people like you on the outcomes that will help us do our next study in evaluating the balance between benefits and harms of statins.

You are selected to participate in this study with a predesigned procedure; i.e., individuals coming to this hospital and currently taking statins or aged at least 40 years old with no history of heart and vascular diseases are eligible to the study. Once you have consented, we only need your responses to questions that we have prepared. Your participation status is not related with any medical care services you will receive. In addition, participation is entirely voluntary and you have full right to withdraw before or in the middle of the interview. However, we encourage you to take part in the study, as your responses would be essential to improve decision making in prescribing statins for preventing future heart and vascular diseases.

I will be interviewing you about your preferences on predefined health problems in the questionnaire. There is not any question that acquires your identifiers. Your data will be kept confidential and used for the mere purpose of the study. The estimated time to complete the interview is 45 minutes. For the time you spend, we will provide you a mobile card that worth 50 ETB.

On behalf of the research team, I would like to thank you in advance for giving me valid responses and contributing your information to improve health of individuals.

**[NAME OF RESPONDENT]**, do you have any question or unclear issues that need further elaborations?

Are you willing to participate in the study?

- *Yes*
- *No*

***For interviewer****:*

*If Yes, acknowledge the respondent and proceed to the next page If No, acknowledge the respondent and quit the interview*

*Interviewer’s Name: ________________________ Date: ___/ __/2016*

Starting time: ____ / ____

mm hh

*Hospital name: _______________________*

***Reminder****: Please do not deviate from the lay descriptions of the health problems beyond or below how they are stated; yet, you can elaborate what the descriptions mean when a need arises.*

# SECTION 1 – DESCRIPTION OF HEALTH PROBLEMS AND SCALING

*Now let’s try to understand lay descriptions of the health problems related to statin therapy. Please pay attention when I read the descriptions or have a look at them carefully. Most of these health problems can have a range of severity from a more to a less severe, but please try to depend only on the described scenario when you answer the questions. When you give me your responses, please try to sympathize into yourself as if you have the health problems. The descriptions are companied with certain pictures. However, please do not provide your responses based on your impression merely on the pictures as the pictures do not entirely express all the clinical features of the health problems.*

*Next, I will ask you to plot (or tell me to plot) on the scale based on how serious the health problem is to you before we proceed to the best-worst scaling. I need to know where does your thought about the seriousness of the health problem lie on the scale. The max value (10) implies the health problem is serious and the minimum value (0) implies that the health problem is the least serious to you.*

*(Interviewer: Please put mark* ***X*** *on the applicable point)*


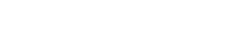

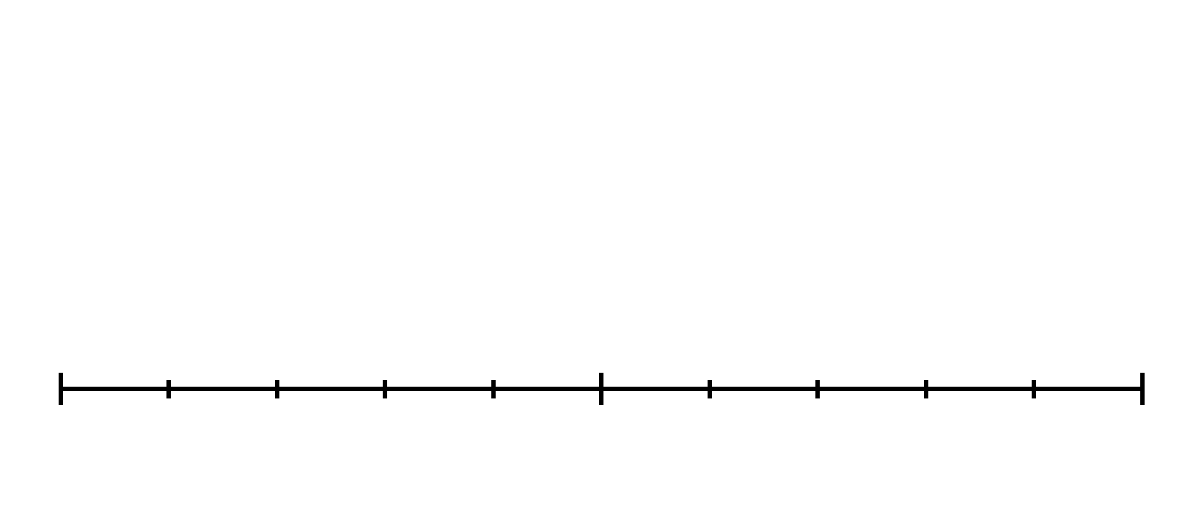

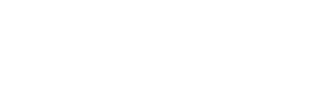


**Chest Pain**

*You have new chest tightness with squeezing pain that occurs with moderate physical activity, such as walking for more than half a kilometer or walk 10-15 minutes on level ground. After a brief rest, the pain goes away, but it comes again when you have similar activity. Your doctor tells you that you don’t have heart attack, but your vessels of your heart are a little tight. You get painkillers and take few other medications and are counseled to adjust your diet and do regular physical activities.*

How serious do you think this health problem is?

**0**

**1**

**2**

**3**

**4**

**5**

**6**

**7**

**8**

**9**

**10**

**Least** serious

**Most** serious


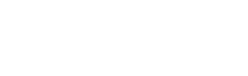

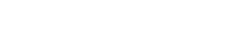

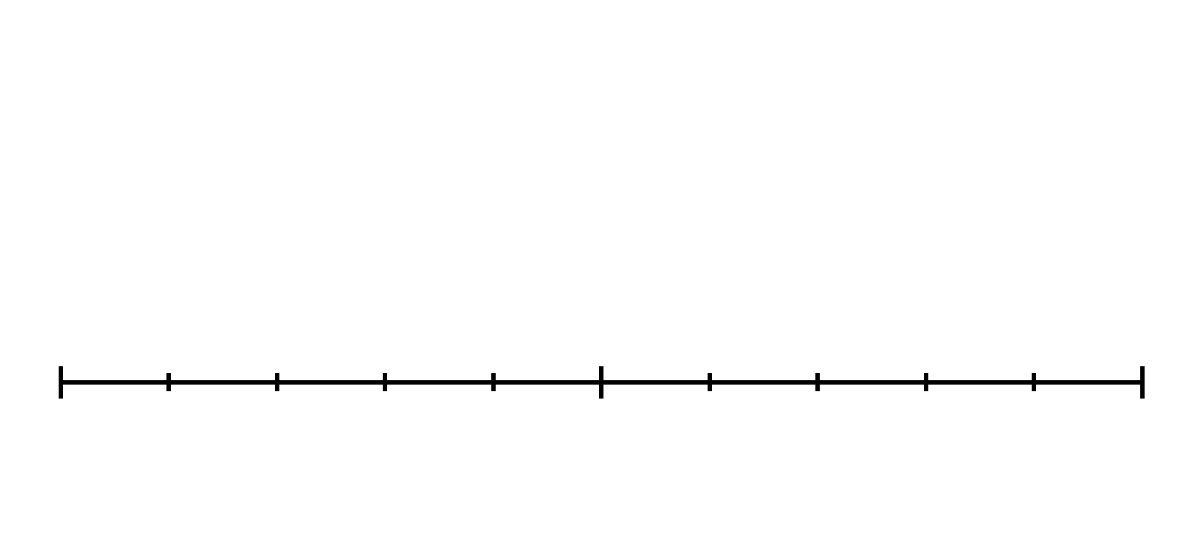

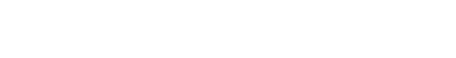


**Moderate Heart Attack**

*Suddenly you have chest pain, feel short of breath and nausea. You are very worried and call an ambulance. After taking medications and care for a few days in a hospital, you return home. You now take a number of new medicines and are making changes to your diet and exercising regularly. If you don’t comply with the treatments and lifestyle adjustments counseled, you are at a higher risk of getting another heart attack again.*

How serious do you think this health problem is?

**0**

**1**

**2**

**3**

**4**

**5**

**6**

**7**

**8**

**9**

**10**

**Least** serious **Most** serious

2

**Least serious**


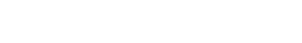

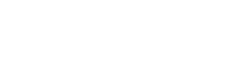

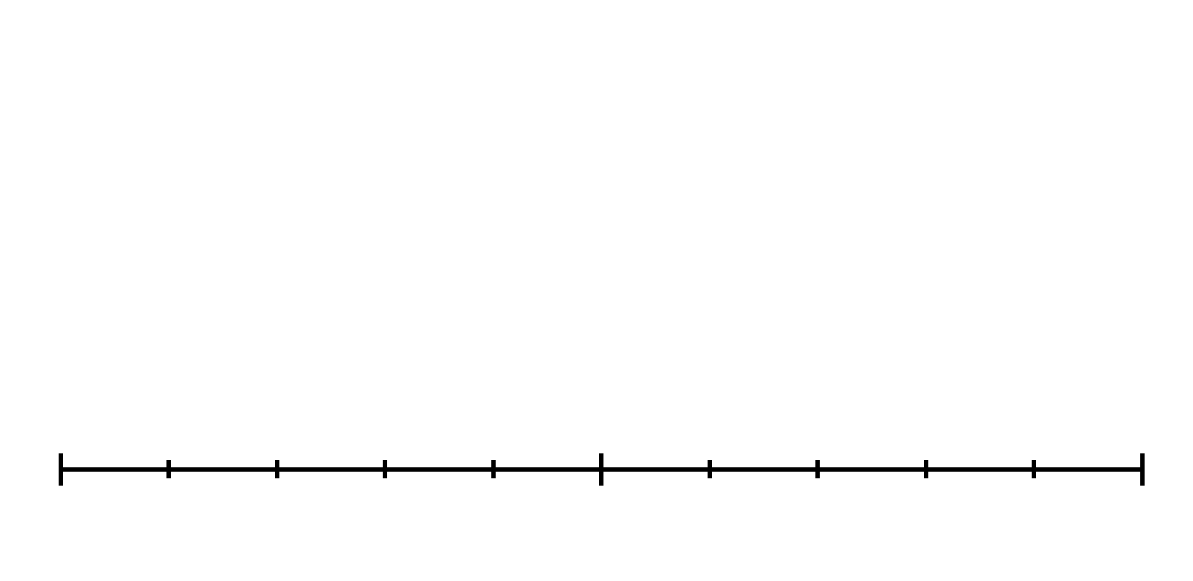

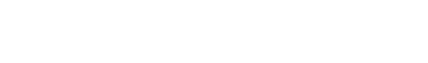


**Severe Heart Attack**

*Your blood flow stops to a part of your heart causing damage to its*

*muscle. Suddenly you have got chest pain that radiates to your shoulder, neck or jaw, are unable to breath and feel faint. A family member calls an ambulance. You get immediate resuscitation and examinations and your doctor does heart surgery. With these interventions, your doctor tells you to go home after your stay at hospital for about two weeks. You are counseled to make changes in diet and exercising regularly. If you don’t comply with the treatments and lifestyle adjustments, you are at a very high risk of getting another heart attack again.*

How serious do you think this health problem is?

**0**

**1**

**2**

**3**

**4**

**5**

**6**

**7**

**8**

**9**

**10**

**Most** serious


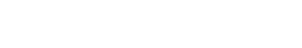

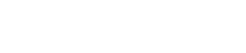

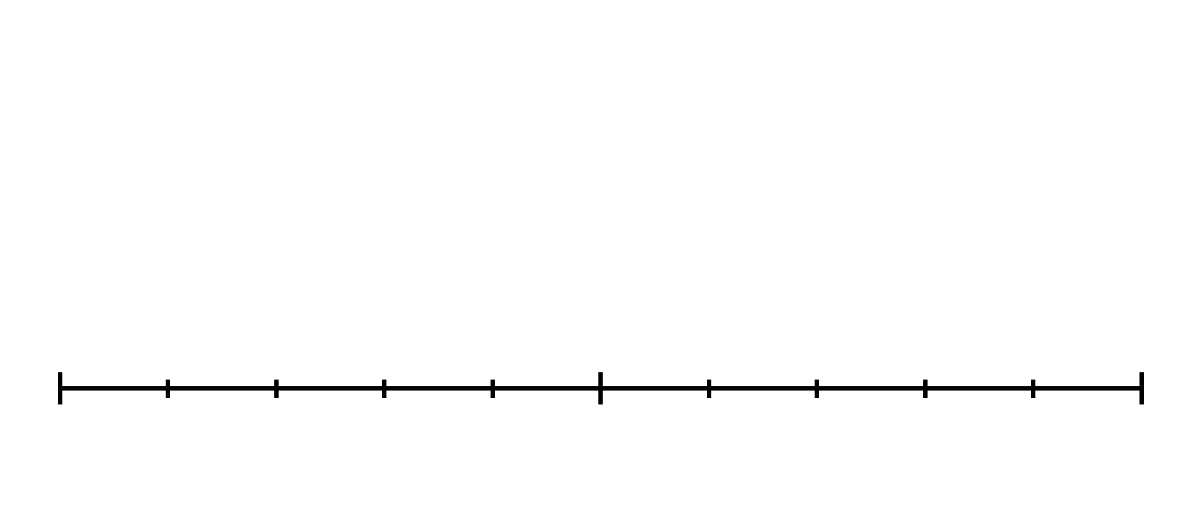

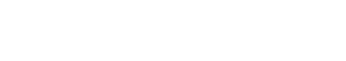


**Moderate Stroke**

*You have sudden weakness in your left arm, difficulty of walking and are slurring your words. A family member calls an ambulance. You spend a number of days in hospital, get new medications and then are moved to a rehabilitation facility for another 4 weeks. Your left arm is better than before but your speech is still a little bit slurred. If you comply with the drugs, and physical exercises, you don’t feel any consequences of the stroke after 4 months.*

How serious do you think this health problem is?

**2**

**3**

**4**

**5**

**6**

**7**

**8**

**Most 9**serious**10**

**Least serious**


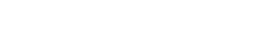

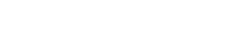

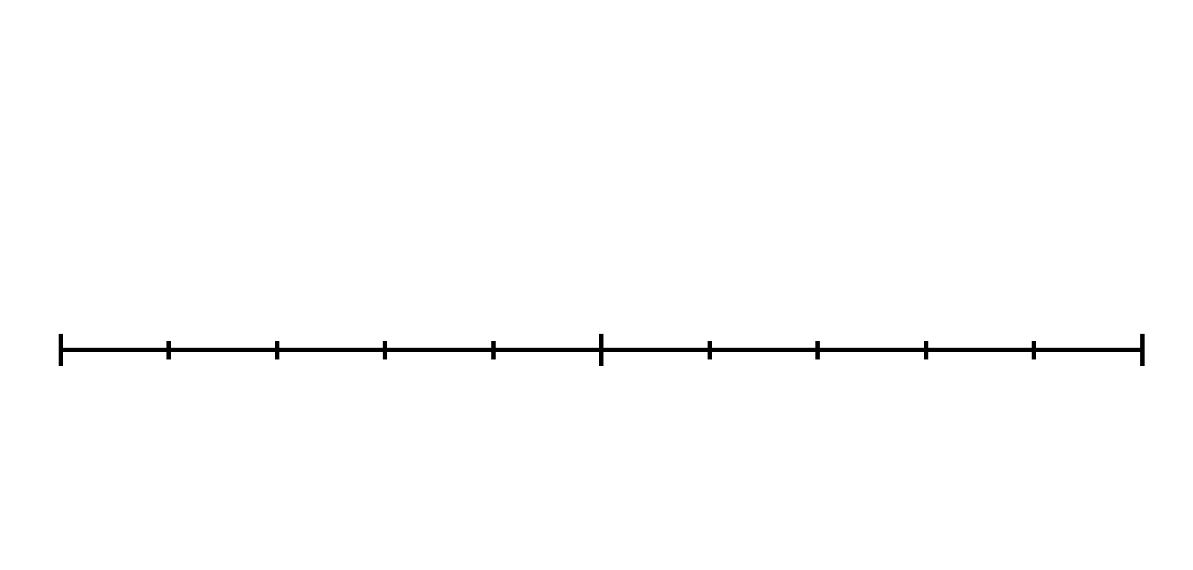

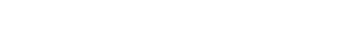


**Severe Stroke**

*You have suddenly got to coma that is risky for death unless treated immediately in a hospital. After taking some medications, you may still be unable to walk and are confined to bed or a wheelchair, have difficulty speaking and depend on others for feeding, toileting and dressing. You have still difficulties to think clearly and remember things. After your discharges from the hospital, you will live with these problems.*

How serious do you think this health problem is?

**0**

**1**

**2**

**3**

**4**

**5**

**6**

**7**

**8**

**9**

**10**

**Least** serious

Most serious

**0**

**1**


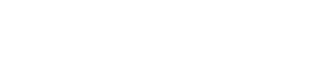

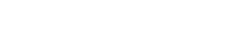

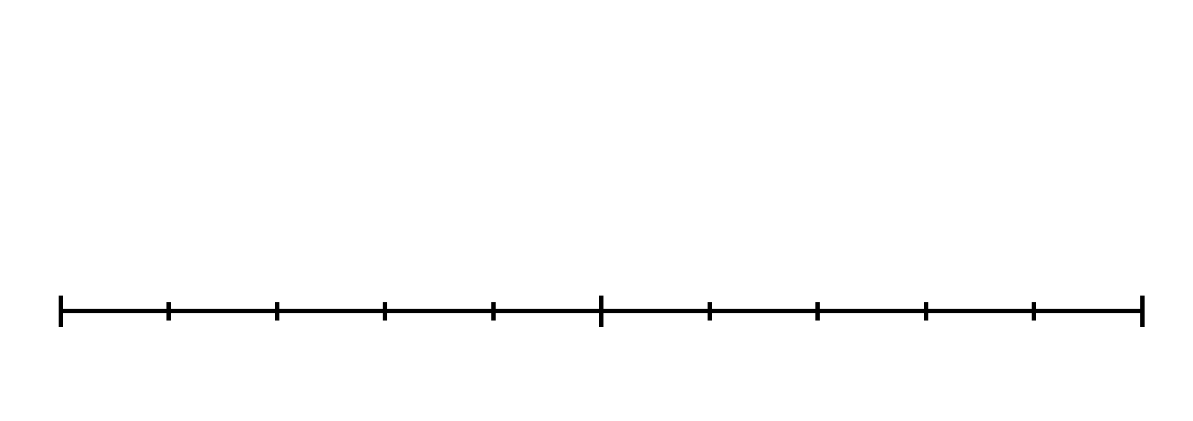

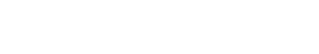


**Heart Failure**

*Your heart doesn’t pump blood as well as it should. You feel tired, are short of breath, especially during the night and your legs are swollen. You need to take several new drugs. You have to go the bathroom many times during the day and at night. If you comply with the treatments, these problems will stay but are tolerable.*

How serious do you think this health problem is?

**0**

**1**

**2**

**3**

**4**

**5**

**6**

**7**

**8**

**9**

**10**

**Least serious Most** serious


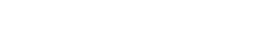

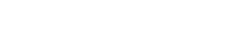

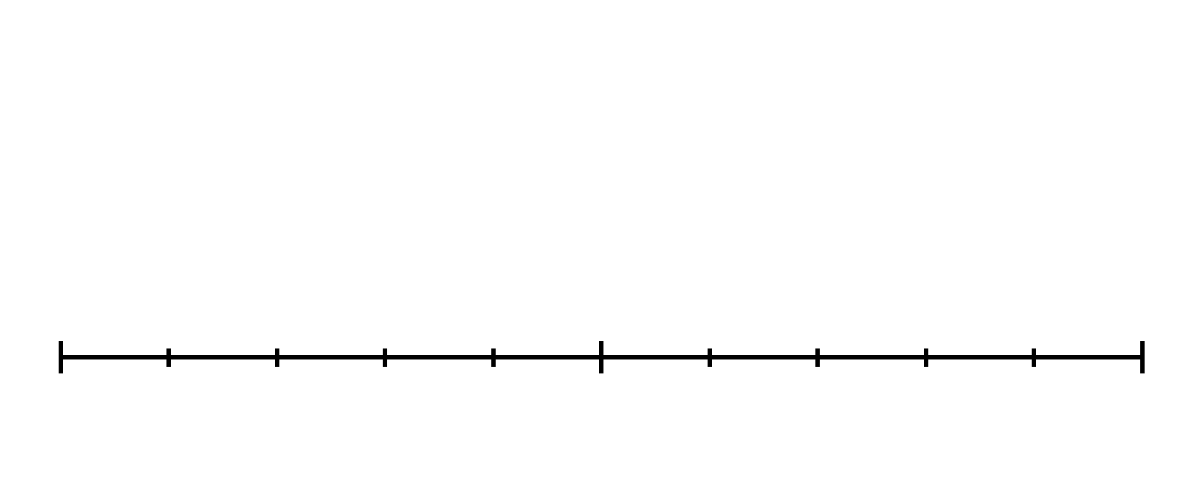

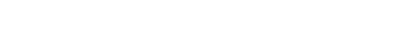


**Acute Kidney failure**

*The function of your kidney is decreased and can’t do their job of removing waste products from your blood properly. You feel quite unwell and nauseated, so tired and short of breath. Your doctor figured out the reasons for the renal failure is a drug. After dropping the drug and 5 days treatment in a hospital, your kidneys work as before and your health is recovered.*

How serious do you think this health problem is?

**Least** serious

**0 1 2 3**

**4**

**5**

**6**

**7**

**8**

**Most** serious

**9 10**

**Liver injury**


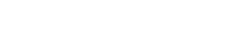

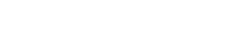

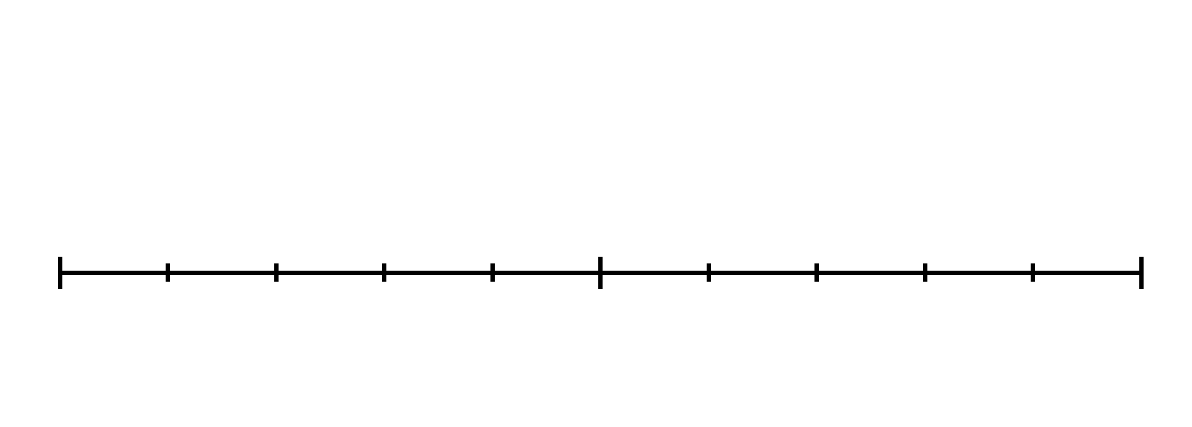


*You have pain on your upper right abdominal part with nausea, vomiting, fatigue, and itching. You may have yellow skin or white outer layer of your eyeball. Your doctor says these problems come from a drug. You don’t take the drug and the problems are gone within three weeks.*

How serious do you think this health problem is?

**0**

**1**

**2**

**3**

**4**

**5**

**6**

**7**

**8**

**9**

**10**

**Least** serious

**Most** serious


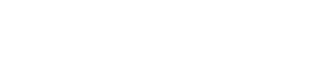


**Muscle pain**


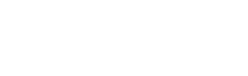

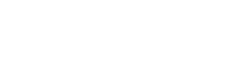

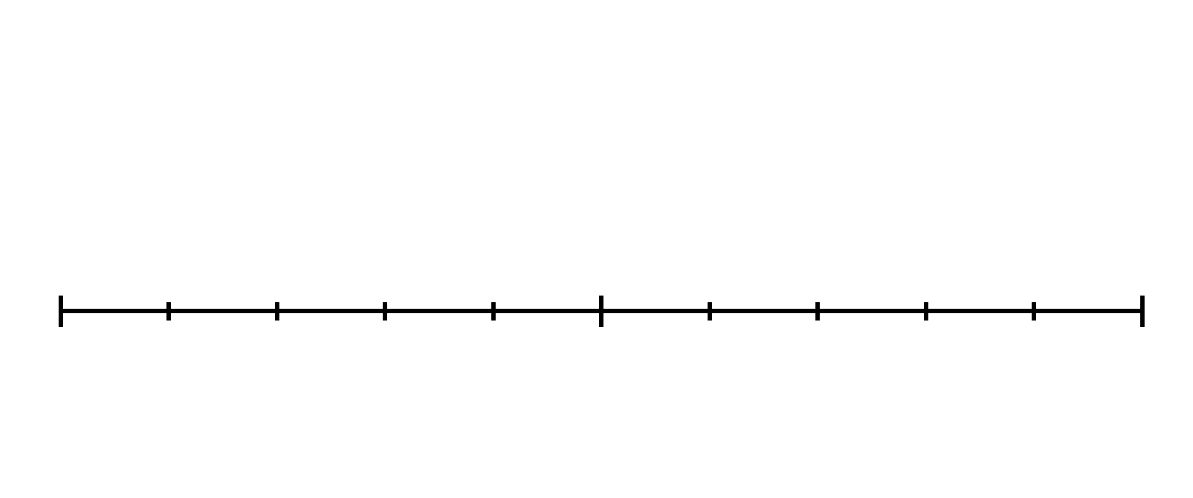


*You are taking a drug every day that is important for your health. After a few weeks, you have muscle weakness on your shoulders, upper arms and thighs. Sometimes you feel cramps, stiffness, and pain in these parts of your body and extremities. The muscle pain comes from a drug. You may not need to go to a hospital but take oral painkillers regularly.*

How serious do you think this health problem is?

**0**

**1**

**2**

**3**

**4**

**5**

**6**

**7**

**8**

**9**

**10**

**Least** serious

**Most** serious


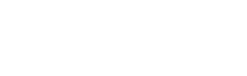

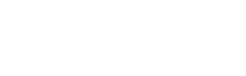

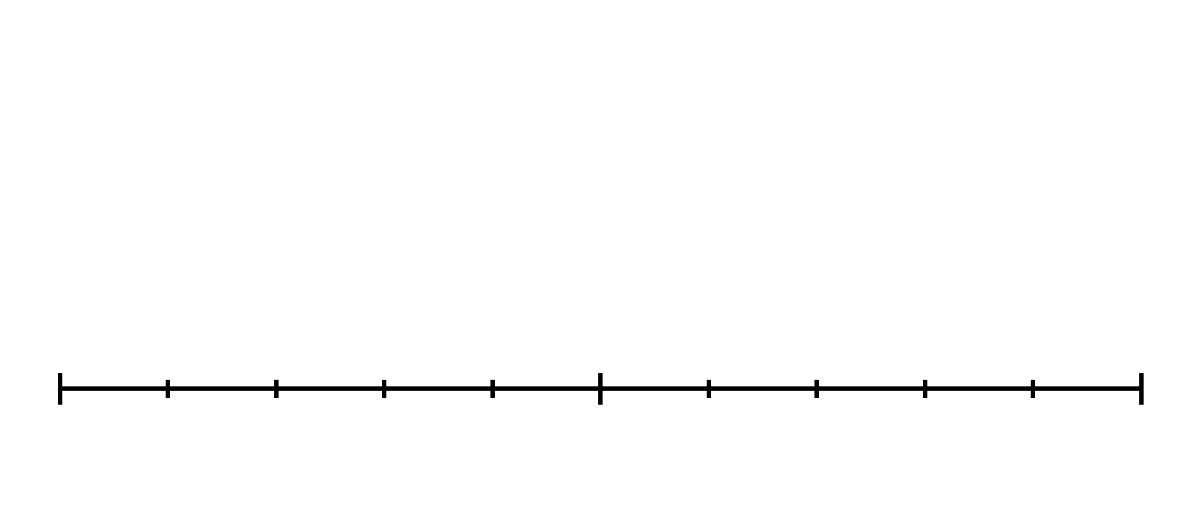

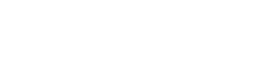


**Cancer**

*You are diagnosed with cancer. You are conscious that the cancer will lower your length of life. You are getting powerful medications as treatment for your cancer one or two days regularly in 6 months. You often feel very fatigued and are worried about your health. You lose weight and your appetite is poor. After you complete your treatment in 6 months, the cancer is not detectable any more, but you are not guaranteed that it comes back in your future.*

How serious do you think this health problem is?

**Least** serious

**0**

**1**

**2**

**3**

**4**

**5**

**6**

**7**

**8**

**Most** serious

**9**

**10**


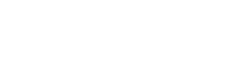

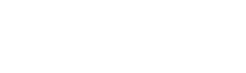

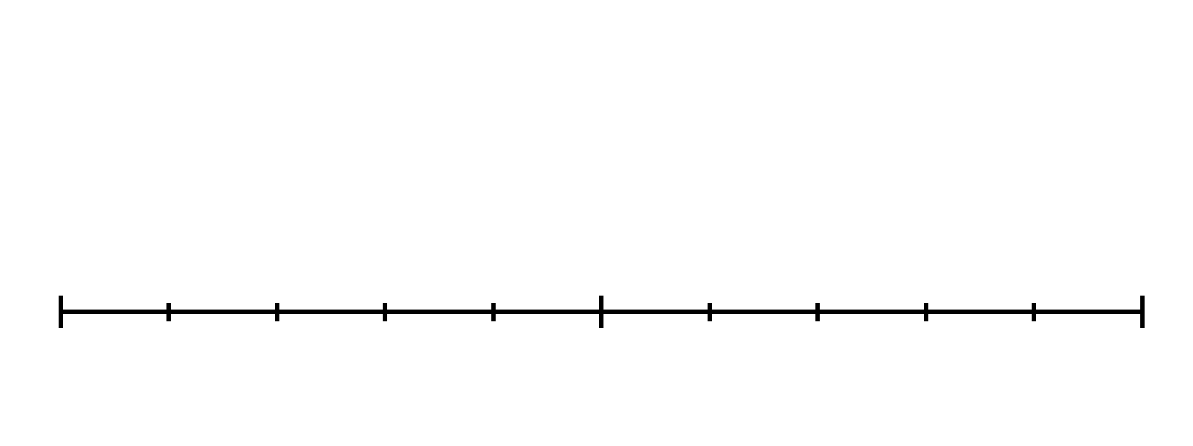

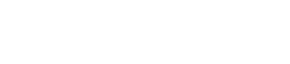


**Side Effects**

*You are taking a drug every day that is important for your health but after two weeks you have such severe side effects (for example, muscle pain, depressive symptoms or nausea) that you decide not take the pills anymore. A week later after dropping the drug, you don’t have problems anymore.*

How serious do you think this health problem is?

**Least** serious

**0 1 2 3**

**4**

**5**

**6**

**7**

**8**

**Most** serious

**9 10**


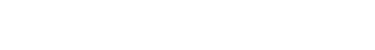


**Sugar disease**


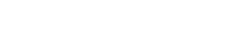

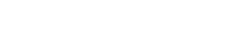

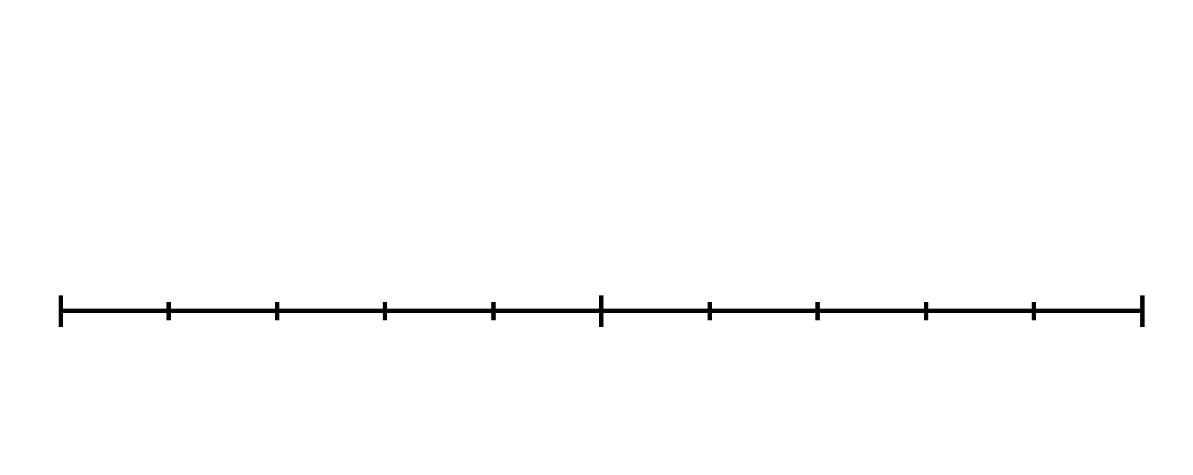


*You have sugar disease, that’s blood sugar level rises higher than normal. You feel increased hunger and are tired, and may need to go to bathroom frequently. You see your doctor and are prescribed drugs that you need to take for the rest of your life to prevent long-term complications. You are counseled to make changes in your lifestyle and monitor your blood sugar.*

How serious do you think this health problem is?

**0**

**1**

**2**

**3**

**4**

**5**

**6**

**7**

**8**

**9**

**10**

**Least** serious

**Most** serious


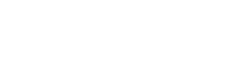

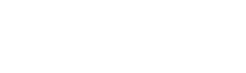

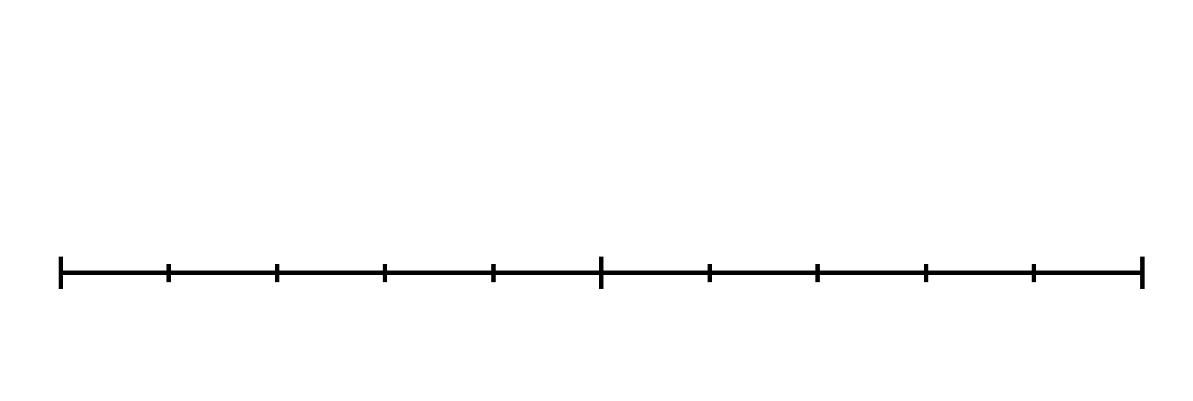

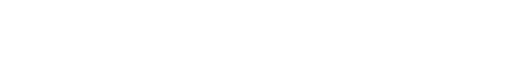


**Nausea and Headache**

*You are taking a drug that is important for your health. After 2 weeks, you start to feel headache and feel like you need to vomit usually once a day. Your doctor says that these problems come from the drug. You stop taking the drug and after 5 days, you don’t feel any head and nausea anymore.*

How serious do you think this health problem is?

**0**

**1**

**2**

**3**

**4**

**5**

**6**

**7**

**8**

**9**

**10**

**Least** serious

**Most** serious

# SECTION 2 – PREFERENCE QUESTIONS

Now I would like to ask you to choose your preferences from set of health problems. There are no right or wrong answers. We want to know how people like you feel about the health problems. *(Interviewer: Please put mark* x *on the applicable point)*

**Q2.1** Among the following problems, which would worry you most and which would worry you least if they were happened to you?

| **Most worrisome**  (Choose one) | **Health problems** | **Least worrisome**  (Choose one) |
| --- | --- | --- |
| ¨ | Moderate stroke | ¨ |
| ¨ | Heart failure | ¨ |
| ¨ | Moderate heart attack | ¨ |
| ¨ | Cancer | ¨ |

**Q2.2** Among the following problems, which would worry you most and which would worry you least if they were happened to you?

| **Most worrisome**  (Choose one) | **Health problems** | **Least worrisome**  (Choose one) |
| --- | --- | --- |
| ¨ | Chest pain | ¨ |
| ¨ | Side Effects | ¨ |
| ¨ | Liver injury | ¨ |
| ¨ | Heart failure | ¨ |

**Q2.3** Among the following problems, which would worry you most and which would worry you least if they were happened to you?

| **Most worrisome**  (Choose one) | **Health problems** | **Least worrisome**  (Choose one) |
| --- | --- | --- |
| ¨ | Liver injury | ¨ |
| ¨ | Muscle pain | ¨ |
| ¨ | Cancer | ¨ |
| ¨ | Sugar disease | ¨ |

**Q2.4** Among the following problems, which would worry you most and which would worry you least if they were happened to you?

| **Most worrisome**  (Choose one) | **Health problems** | **Least worrisome**  (Choose one) |
| --- | --- | --- |
| ¨ | Acute kidney failure | ¨ |
| ¨ | Cancer | ¨ |
| ¨ | Nausea/headache | ¨ |
| ¨ | Side Effects | ¨ |

**Q2.5** Among the following problems, which would worry you most and which would worry you least if they were happened to you?

| **Most worrisome**  (Choose one) | **Health problems** | **Least worrisome**  (Choose one) |
| --- | --- | --- |
| ¨ | Cancer | ¨ |
| ¨ | Chest pain | ¨ |
| ¨ | Severe stroke | ¨ |
| ¨ | Severe heart attack | ¨ |

**Q2.6** Among the following problems, which would worry you most and which would worry you least if they were happened to you?

| **Most worrisome**  (Choose one) | **Health problems** | **Least worrisome**  (Choose one) |
| --- | --- | --- |
| ¨ | Moderate heart attack | ¨ |
| ¨ | Acute kidney failure | ¨ |
| ¨ | Chest pain | ¨ |
| ¨ | Muscle pain | ¨ |

**Q2.7** Among the following problems, which would worry you most and which would worry you least if they were happened to you?

| **Most worrisome**  (Choose one) | **Health problems** | **Least worrisome**  (Choose one) |
| --- | --- | --- |
| ¨ | Side Effects | ¨ |
| ¨ | Moderate heart attack | ¨ |
| ¨ | Sugar diseases | ¨ |
| ¨ | Severe stroke | ¨ |

**Q2.8** Among the following problems, which would worry you most and which would worry you least if they were happened to you?

| **Most worrisome**  (Choose one) | **Health problems** | **Least worrisome**  (Choose one) |
| --- | --- | --- |
| ¨ | Sugar diseases | ¨ |
| ¨ | Nausea/headache | ¨ |
| ¨ | Moderate stroke | ¨ |
| ¨ | Chest pain | ¨ |

**Q2.9** Among the following problems, which would worry you most and which would worry you least if they were happened to you?

| **Most worrisome**  (Choose one) | **Health problems** | **Least worrisome**  (Choose one) |
| --- | --- | --- |
| ¨ | Nausea/headache | ¨ |
| ¨ | Liver injury | ¨ |
| ¨ | Severe heart attack | ¨ |
| ¨ | Moderate heart attack | ¨ |

**Q2.10** Among the following problems, which would worry you most and which would worry you least if they were happened to you?

| **Most worrisome**  (Choose one) | **Health problems** | **Least worrisome**  (Choose one) |
| --- | --- | --- |
| ¨ | Heart failure | ¨ |
| ¨ | Severe stroke | ¨ |
| ¨ | Muscle pain | ¨ |
| ¨ | Nausea/headache | ¨ |

**Q2.11** Among the following problems, which would worry you most and which would worry you least if they were happened to you?

| **Most worrisome**  (Choose one) | **Health problems** | **Least worrisome**  (Choose one) |
| --- | --- | --- |
| ¨ | Severe heart attack | ¨ |
| ¨ | Sugar disease | ¨ |
| ¨ | Heart failure | ¨ |
| ¨ | Acute kidney failure | ¨ |

**Q2.12** Among the following problems, which would worry you most and which would worry you least if they were happened to you?

| **Most worrisome**  (Choose one) | **Health problems** | **Least worrisome**  (Choose one) |
| --- | --- | --- |
| ¨ | Severe stroke | ¨ |
| ¨ | Moderate stroke | ¨ |
| ¨ | Acute kidney failure | ¨ |
| ¨ | Liver injury | ¨ |

**Q2.13** Among the following problems, which would worry you most and which would worry you least if they were happened to you?

| **Most worrisome**  (Choose one) | **Health problems** | **Least worrisome**  (Choose one) |
| --- | --- | --- |
| ¨ | Muscle pain | ¨ |
| ¨ | Severe heart attack | ¨ |
| ¨ | Side Effects | ¨ |
| ¨ | Moderate stroke | ¨ |

# SECTION 3: BACKGROUND CHARACTERISTICS OF RESPONDENTS

Q3.1 Sex

- Male
- Female

Q3.2 What is your age as of your last birthday?

___________yrs

Q3.3 What are your total years of education?

___________ yrs

Q3.4 What is your current job?

- Salaried
- Private business
- Retired
- No job

¨ Other (specify) _______________

Q3.5 Do you have any medically identified health problem? If yes, please state it/them. *(Interviewer: Please probe the respondent about existing morbidity)*

| ¨ | No |  |
| --- | --- | --- |
| ¨ | Yes |  |
|  | I. | ____________________ III. ____________________ |
|  | II. | ____________________ IV. ____________________ |

Q3.6 Do you take statins?

- No
- Yes, currently
- Yes, previously

Q3.7 For which of the health problems is medical care not available in your area?

Select the first three.

I. _____________________ II. _____________________ III. _____________________

Q3.8 Who do you live with?

- Alone
- Family (partner/child/parent)

¨ Other (specify) __________________

Q3.9 What do you think are your main reasons that influence your preferences of the health problems?

*[Interviewer: Please explain this to the respondents to state their concerns that would make them change their preferences]*

- Availability of the medical care
- Severity of the health problems
- Curability of the health problems
- Long-term consequences
- Affordability of the medical care Others (Specify):

¨ ________________________________

¨ ________________________________

# SECTION 4 RATING OF THE QUESTIONNAIRE

Would you please give me your answer whether you agree with the following statements?

Q4.1 I found it easy to understand the questions

- I strongly agree
- I agree
- I neither agree nor disagree
- I disagree
- I strongly disagree

Q4.2 I found it easy to answer all the questions

- I strongly agree
- I agree
- I neither agree nor disagree
- I disagree
- I strongly disagree

Q4.3 I answered all questions in a way consistent with my preferences

- I strongly agree
- I agree
- I neither agree nor disagree
- I disagree
- I strongly disagree

Yebyo HG, Aschmann HE, Yu T, Puhan MA: Should statin guidelines consider patient preferences? Eliciting preferences of benefit and harm outcomes of statins for primary prevention of cardiovascular disease in the sub-Saharan African and European contexts. BMC cardiovascular disorders. 2018, 18(1):97. doi:10.1186/s12872-018-0838-9

**Best-Worst Questionnaire for STATINS (Persian version)**

**تعیین اولویت انتخاب بیماران در خصوص مصرف یا عدم مصرف داروی استاتین بر اساس منافع و عوارض آن**

**یادآوری:** همکار محترم پژوهش لطفا در توصیف مشکلات سلامت، کمتر یا بیشتر از آنچه که بیان شده است استفاده نشود. با این حال در صورت نیاز شما می توانید شرح دهید که این توضیحات فقط در حد نیاز برای تکمیل این پرسشنامه است و شرکت کنده محترم می تواند برای توضیحات بیشتر بعدا از پزشک سوال نماید.

**بخش اول:**

شرکت کننده محترم، در هنگام خواندن توضیحات و یا نگاه کردن به تصاویر لطفا با دقت توجه کنید. اکثر این مشکلات سلامتی می توانند طیف وسیعی از شدت را (از کمترین تا بیشترین شدت) داشته باشند. هنگام پاسخگویی به سوالات سعی کنید خود را به جای بیمار فرض کنید تا درک بهتری از مشکل داشته باشید و فرض نمایید که اگر شما آن مشکلات سلامت را داشتید پاسخ شما چه بود. در بیان توضیحات از تصاویر خاص آن استفاده شده است با این حال با توجه به اینکه تصاویر بطور کامل بیانگر تمام ابعاد و جنبه های بیماری نمی باشند لطفا پاسخ های شما صرفاً بر اساس درک شما از روی تصاویر نباشد. قبل از اینکه مرحله بعدی شروع شود از شما می خواهیم که بر اساس اینکه آن مشکل سلامت از نظر شما چقدر جدی است آن را بر روی مقیاس خط کش مشخص نمایید. حداکثر این مقیاس (10) می باشد که نشان دهنده بیشترین و حداقل مقدار آن (0) به معنی کمترین است.

1. **درد قفسه سینه**

شما دردی فشار دهنده همراه با گرفتگی در قفسه سینه دارید که به تازگی اتفاق افتاده و با فعالیت بدنی متوسط مانند راه رفتن بیش از نیم کیلومتر، یا 10 تا 15 دقیقه راه رفتن در سطح صاف رخ می دهد. این درد با کمی استراحت موقتاً رفع می شود ولی با شروع مجدد فعالیت دوباره درد ایجاد می شود. پزشک شما را معاینه می کند و می گوید که حمله قلبی ندارید اما رگ های قلب تان کمی تنگ شده اند. برای شما داروهای مسکن تجویز می کند و برای بهبود رژیم غذایی، ورزش و ترک دخانیات مشاوره می دهد.

**بنظر شما این مشکل سلامت چقدر جدی است لطفا بر روی مقیاس زیر آن را مشخص نمایید؟**


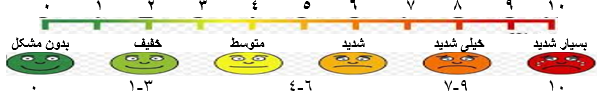


1. **حمله قلبی متوسط:**

شما درد شدید و ناگهانی در قفسه سینه همراه با احساس تنگی نفس و تهوع دارید و با حالت نگران به مرکز اورژانس مراجعه می کنید. شما دچار حمله قلبی شده اید، در بیمارستان بستری شده و تحت نظر قرار می گیرید. پس از مصرف داروها و چند روز مراقبت، از بیمارستان ترخیص شده و به خانه بر می گردید. به شما توصیه می شود که رژیم غذایی و سبک زندگی خود را تغییر و اصلاح کنید و داروهای تجویز شده را منظم مصرف کنید در غیر اینصورت ممکن است مجدداً دچار حمله قلبی دیگری شوید.

**بنظر شما این مشکل سلامت چقدر جدی است لطفا بر روی مقیاس زیر آن را مشخص نمایید؟**


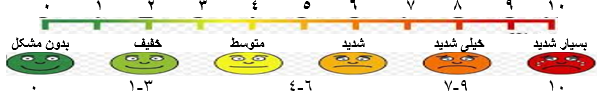


1. **حمله قلبی شدید:**

شما درد ناگهانی و شدید در قفسه سینه دارید که این درد در شانه ها، گردن و فک تان منتشر می شود. در این حالت شما قادر به نفس کشیدن نیستید بصورتیکه احساس مرگ و ضعف می کنید. اعضای خانواده تان سریعا با اورژانس تماس می گیرند و در بیمارستان بستری می شوید. قسمتی از جریان خون قلب تان قطع شده است که باعث آسیب رساندن به عضلات قلب می شود. با معاینه پزشکی و عملیات احیاء (ماساژ و شوک الکتریکی قلب و تنفس مصنوعی) سریعا تحت عمل جراحی قلب قرار می گیرید. پس از حدود دو هفته بستری در بیمارستان، مرخص شده و به خانه برمیگردید. پزشک معالج توصیه می کند که حتماً باید رژیم غذایی و سبک زندگی خود را تغییر داده و اصلاح کنید در غیر اینصورت احتمال حملات قلبی شدید دیگر وجود خواهد داشت.

**بنظر شما این مشکل سلامت چقدر جدی است لطفا بر روی مقیاس زیر آن را مشخص نمایید؟**


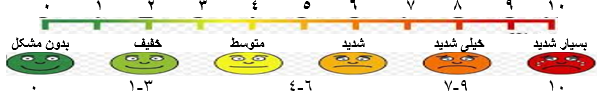


1. **سکته مغزی متوسط**

بطور ناگهانی دچار ضعف در بازوی چپ، مشکل در راه رفتن و بیان کلمات می شوید. اعضای خانواده تان سریعا با اورژانس تماس می گیرند. چند روز در بیمارستان بستری می شوید و داروهای جدید مصرف می کنید. با کاهش علائم اولیه مرخص شده، سپس برای 4 هفته دیگر به یک مرکز توانبخشی منتقل می شوید. کم کم ضعف در بازوی چپ بهتر می شود، اما در صحبت کردن هنوز جملات را کمی نامفهوم بیان می کنید. اگر از داروها و فعالیت های فیزیکی توصیه شده پیروی کنید بعد از 4 ماه هیچ آثاری از سکته مغزی را مشاهده نمی کنید. اما باید در مورد تغییر رژیم غذایی و انجام فعالیت بدنی منظم و ترک دخانیات اقدام کنید در غیر اینصورت احتمال سکته مغزی دیگر وجود خواهد داشت.

**بنظر شما این مشکل سلامت چقدر جدی است لطفا بر روی مقیاس زیر آن را مشخص نمایید؟**


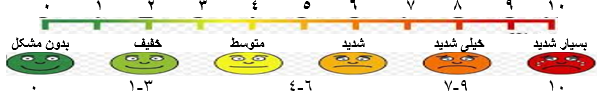


1. **سکته مغزی شدید:**

شما بطور ناگهانی به کما می روید که در آن خطر مرگ وجود دارد، مگر اینکه بلافاصله و فوری در بیمارستان درمان شوید. پس از مصرف داروها ممکن است هنوز قادر به راه رفتن نباشید و به رختخواب یا صندلی چرخدار محدود شوید، درصحبت کردن مشکل داشته باشید و برای غذا خوردن، توالت رفتن و پوشیدن لباس به دیگران وابسته شوید و هنوز هم در فکر کردن و یادآوری مسائل دچار مشکل هستید. در صورت زنده ماندن و پس از ترخیص از بیمارستان، شما تا اخر عمر با این مشکلات زندگی می کنید.

**بنظر شما این مشکل سلامت چقدر جدی است لطفا بر روی مقیاس زیر آن را مشخص نمایید؟**


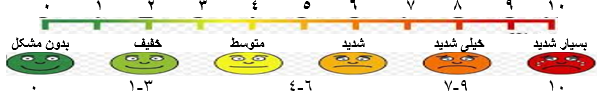


1. **نارسایی قلبی:**

قلب تان، به اندازه کافی خون را پمپ نمی کند. پاهایتان ورم کرده و احساس خستگی می کنید و موقع خوابیدن احساس تنگی نفس دارید. نیاز به مصرف چندین داروی جدید دارید. باید در طول روز و شب چندین بار برای دفع ادرار به توالت بروید. در صورت پیروی از درمان این مشکلات همچنان باقی می مانند، ولی تحمل پذیر هستند.

**بنظر شما این مشکل سلامت چقدر جدی است لطفا بر روی مقیاس زیر آن را مشخص نمایید؟**


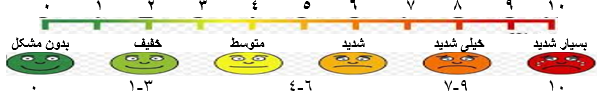


1. **نارسایی حاد کلیه:**

عملکرد کلیه تان کاهش می یابد و نمی تواند کار خود را برای حذف مواد زائد از خون انجام دهد. شما احساس ناخوشی، تهوع و همچنین خستگی و تنگی نفس دارید. دکترتان متوجه می شود که دلیل نارسایی کلیه شما بعلت مصرف دارو است. با قطع مصرف دارو و درمان 5 روزه در بیمارستان، عملکرد کلیه ها به حالت طبیعی برگشته و سلامتی شما بهبود می یابد.

**بنظر شما این مشکل سلامت چقدر جدی است لطفا بر روی مقیاس زیر آن را مشخص نمایید؟**


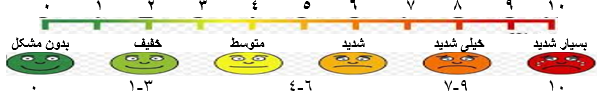


1. **آسیب کبدی:**

در قسمت بالای سمت راست شکم و زیر دنده ها، احساس درد همراه با تهوع، استفراغ، خستگی و خارش بدن دارید. همچنین ممکن است سفیدی چشم یا برخی نقاط پوست شما زرد رنگ شده باشد. پزشک به شما می گوید که این مشکلات به دلیل مصرف دارو است. با قطع دارو در مدت سه هفته مشکلات رفع می شود.

**بنظر شما این مشکل سلامت چقدر جدی است لطفا بر روی مقیاس زیر آن را مشخص نمایید؟**


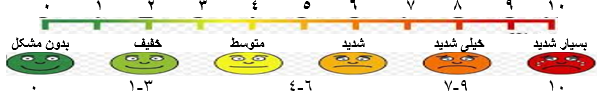


1. **درد عضلانی:**

چند هفته پس از مصرف دارویی که روزانه مصرف می کنید دچار ضعف و درد عضلانی در شانه، بازوها و ران ها می شوید. که گاهی با گرفتگی، سفتی و درد در این قسمت ها همراه است. درد عضلانی ناشی از دارو می باشد. این درد بدون بستری در بیمارستان و با مصرف داروی مسکن بهتر می شوید.

**بنظر شما این مشکل سلامت چقدر جدی است لطفا بر روی مقیاس زیر آن را مشخص نمایید؟**


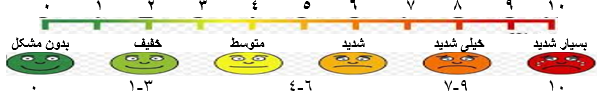


1. **سرطان:**

پزشک تشخیص داده شما مبتلا به سرطان شده اید که طول عمر شما را کم می کند. برای درمان سرطان تان هر روز یا یک روز درمیان به مدت 6 ماه از داروهای قوی استفاده می‌کنید. اغلب احساس خستگی می کنید و در مورد سلامت خود نگران هستید. دچار کاهش وزن می شوید و اشتهای تان کم می شود. پس از تکمیل درمان در طول 6 ماه، علائم سرطان از بین رفته و قابل مشاهده نیستد ولی از بازگشت آن نگران می باشید.

**بنظر شما این مشکل سلامت چقدر جدی است لطفا بر روی مقیاس زیر آن را مشخص نمایید؟**


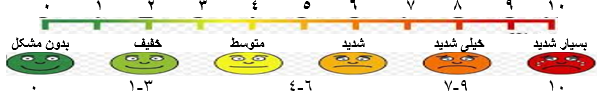


1. **اثرات و عوارض جانبی دارویی:**

به صورت روزانه دارویی مصرف می کنید که آن دارو برای سلامت تان مهم است. اما بعد از دو هفته از مصرف آن دارو دچار عوارض جانبی شدید (مانند علائم افسردگی و اختلالات خواب) می شوید که خودتان تصمیم می گیرید آن دارو را دیگر مصرف نکنید. یک هفته پس از قطع دارو، دیگر مشکلی ندارید.

**بنظر شما این مشکل سلامت چقدر جدی است لطفا بر روی مقیاس زیر آن را مشخص نمایید؟**


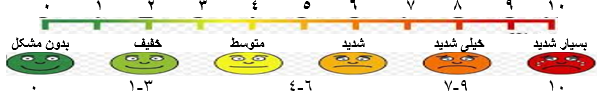


1. **دیابت نوع دو/ بیماری قند:**

بیماری قند دارید که در آن سطح قند خون شما بالاتر از حد طبیعی است. احساس گرسنگی و تشنگی بیشتری می‌کنید و خسته هستید و ممکن است مجبور باشید به طور مرتب برای دفع ادرار به دستشویی بروید. به پزشک مراجعه می کنید و پزشک به منظور جلوگیری از عوارض طولانی مدت این بیماری داروهایی را تجویز می کند که برای بقیه زندگی خود به آن نیاز دارید. همچنین در مورد تغییر در شیوه زندگی به خصوص رژیم غذایی و فعالیت بدنی مناسب و کنترل و پایش قند خون به شما مشاوره می دهد و توصیه هایی می کند که باید آنها را رعایت کنید.

**بنظر شما این مشکل سلامت چقدر جدی است لطفا بر روی مقیاس زیر آن را مشخص نمایید؟**


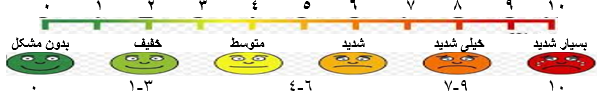


1. **تهوع و سردرد:**

دارویی مصرف می کنید که آن دارو برای سلامت شما مهم است. اما بعد از دو هفته از مصرف آن دارو دچار سردرد می شوید و معمولا در حدود یک بار در روز هم احساس تهوع و گاهی استفراغ می‌کنید. دکتر به شما می گوید این مشکلات به خاطر مصرف دارو است و شما مصرف دارو را قطع می‌کنید که 5 روز پس از قطع مصرف دارو دیگر هیچ گونه احساس سردرد و حالت تهوع ندارید.

**بنظر شما این مشکل سلامت چقدر جدی است لطفا بر روی مقیاس زیر آن را مشخص نمایید؟**


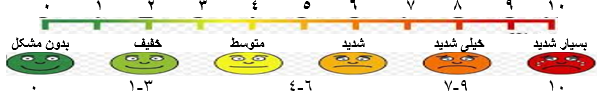


**بخش دوم: پرسشنامه PREFERENCE**

اکنون می خواهیم که شما ترجیحات خود را از مجموعه ای از مشکلات سلامتی که در هر سری ارائه می شود را انتخاب کنید. هیچ جواب درست یا غلطی وجود ندارد. ما می خواهیم احساس افراد مشابه شما در مورد مشکلات سلامت را بدانیم.

مصاحبه کننده: لطفا علامت X را در نقطه مناسب قرار دهید.

| سری 1 (از میان مشکلات زیر، کدامیک اگر برای شما رخ دهد بیشتر نگران و کدامیک رخ دهد کمتر نگران می شوید؟) | | |
| --- | --- | --- |
| **بیشترین نگرانی**(تنها یک گزینه انتخاب شود) | مشکلات سلامت | **کمترین نگرانی**(تنها یک گزینه انتخاب شود) |
| ⬜ | **سکته مغزی متوسط** | ⬜ |
| ⬜ | **نارسایی قلبی** | ⬜ |
| ⬜ | **حمله قلبی متوسط** | ⬜ |
| ⬜ | **سرطان** | ⬜ |

| سری 2 (از میان مشکلات زیر، کدامیک اگر برای شما رخ دهد بیشتر نگران و کدامیک رخ دهد کمتر نگران می شوید؟) | | |
| --- | --- | --- |
| **بیشترین نگرانی**(تنها یک گزینه انتخاب شود) | مشکلات سلامت | **کمترین نگرانی**(تنها یک گزینه انتخاب شود) |
| ⬜ | **درد قفسه سینه** | ⬜ |
| ⬜ | **اثرات جانبی** | ⬜ |
| ⬜ | **آسیب کبدی** | ⬜ |
| ⬜ | **نارسایی قلبی** | ⬜ |

| سری 3 (از میان مشکلات زیر، کدامیک اگر برای شما رخ دهد بیشتر نگران و کدامیک رخ دهد کمتر نگران می شوید؟) | | |
| --- | --- | --- |
| **بیشترین نگرانی**(تنها یک گزینه انتخاب شود) | مشکلات سلامت | **کمترین نگرانی**(تنها یک گزینه انتخاب شود) |
| ⬜ | **آسیب کبدی** | ⬜ |
| ⬜ | **درد عضلانی** | ⬜ |
| ⬜ | **سرطان** | ⬜ |
| ⬜ | **دیابت نوع دو/بیماری قند** | ⬜ |

| سری 4 (از میان مشکلات زیر، کدامیک اگر برای شما رخ دهد بیشتر نگران و کدامیک رخ دهد کمتر نگران می شوید؟) | | |
| --- | --- | --- |
| **بیشترین نگرانی**(تنها یک گزینه انتخاب شود) | مشکلات سلامت | **کمترین نگرانی**(تنها یک گزینه انتخاب شود) |
| ⬜ | **نارسایی حاد کلیوی** | ⬜ |
| ⬜ | **سرطان** | ⬜ |
| ⬜ | **تهوع و سردرد** | ⬜ |
| ⬜ | **اثرات جانبی** | ⬜ |

| سری 5 (از میان مشکلات زیر، کدامیک اگر برای شما رخ دهد بیشتر نگران و کدامیک رخ دهد کمتر نگران می شوید؟) | | |
| --- | --- | --- |
| **بیشترین نگرانی**(تنها یک گزینه انتخاب شود) | مشکلات سلامت | **کمترین نگرانی**(تنها یک گزینه انتخاب شود) |
| ⬜ | **سرطان** | ⬜ |
| ⬜ | **درد قفسه سینه** | ⬜ |
| ⬜ | **سکته مغزی شدید** | ⬜ |
| ⬜ | **حمله قلبی شدید** | ⬜ |

| سری 6 (از میان مشکلات زیر، کدامیک اگر برای شما رخ دهد بیشتر نگران و کدامیک رخ دهد کمتر نگران می شوید؟) | | |
| --- | --- | --- |
| **بیشترین نگرانی**(تنها یک گزینه انتخاب شود) | مشکلات سلامت | **کمترین نگرانی**(تنها یک گزینه انتخاب شود) |
| ⬜ | **حمله قلبی متوسط** | ⬜ |
| ⬜ | **نارسایی حاد کلیوی** | ⬜ |
| ⬜ | **درد قفسه سینه** | ⬜ |
| ⬜ | **درد عضلانی** | ⬜ |

| سری 7 (از میان مشکلات زیر، کدامیک اگر برای شما رخ دهد بیشتر نگران و کدامیک رخ دهد کمتر نگران می شوید؟) | | |
| --- | --- | --- |
| **بیشترین نگرانی**(تنها یک گزینه انتخاب شود) | مشکلات سلامت | **کمترین نگرانی**(تنها یک گزینه انتخاب شود) |
| ⬜ | **اثرات جانبی** | ⬜ |
| ⬜ | **حمله قلبی متوسط** | ⬜ |
| ⬜ | **دیابت نوع دو/ بیماری قند** | ⬜ |
| ⬜ | **سکته مغزی شدید** | ⬜ |

| سری 8 (از میان مشکلات زیر، کدامیک اگر برای شما رخ دهد بیشتر نگران و کدامیک رخ دهد کمتر نگران می شوید؟) | | |
| --- | --- | --- |
| **بیشترین نگرانی**(تنها یک گزینه انتخاب شود) | مشکلات سلامت | **کمترین نگرانی**(تنها یک گزینه انتخاب شود) |
| ⬜ | **دیابت نوع دو/ بیماری قند** | ⬜ |
| ⬜ | **تهوع و سردرد** | ⬜ |
| ⬜ | **سکته مغزی متوسط** | ⬜ |
| ⬜ | **درد قفسه سینه** | ⬜ |

| سری 9 (از میان مشکلات زیر، کدامیک اگر برای شما رخ دهد بیشتر نگران و کدامیک رخ دهد کمتر نگران می شوید؟) | | |
| --- | --- | --- |
| **بیشترین نگرانی**(تنها یک گزینه انتخاب شود) | مشکلات سلامت | **کمترین نگرانی**(تنها یک گزینه انتخاب شود) |
| ⬜ | **تهوع و سردرد** | ⬜ |
| ⬜ | **آسیب کبدی** | ⬜ |
| ⬜ | **حمله قلبی شدید** | ⬜ |
| ⬜ | **حمله قلبی متوسط** | ⬜ |

| سری10 (از میان مشکلات زیر، کدامیک اگر برای شما رخ دهد بیشتر نگران و کدامیک رخ دهد کمتر نگران می شوید؟) | | |
| --- | --- | --- |
| **بیشترین نگرانی**(تنها یک گزینه انتخاب شود) | مشکلات سلامت | **کمترین نگرانی**(تنها یک گزینه انتخاب شود) |
| ⬜ | **نارسایی قلبی** | ⬜ |
| ⬜ | **سکته مغزی شدید** | ⬜ |
| ⬜ | **درد عضلانی** | ⬜ |
| ⬜ | **تهوع و سردرد** | ⬜ |

| سری 11 (از میان مشکلات زیر، کدامیک اگر برای شما رخ دهد بیشتر نگران و کدامیک رخ دهد کمتر نگران می شوید؟) | | |
| --- | --- | --- |
| **بیشترین نگرانی**(تنها یک گزینه انتخاب شود) | مشکلات سلامت | **کمترین نگرانی**(تنها یک گزینه انتخاب شود) |
| ⬜ | **حمله قلبی شدید** | ⬜ |
| ⬜ | **دیابت نوع دو/بیماری قند** | ⬜ |
| ⬜ | **نارسایی قلبی** | ⬜ |
| ⬜ | **نارسایی حاد کلیوی** | ⬜ |

| سری 12(از میان مشکلات زیر، کدامیک اگر برای شما رخ دهد بیشتر نگران و کدامیک رخ دهد کمتر نگران می شوید؟) | | |
| --- | --- | --- |
| **بیشترین نگرانی**(تنها یک گزینه انتخاب شود) | مشکلات سلامت | **کمترین نگرانی**(تنها یک گزینه انتخاب شود) |
| ⬜ | **سکته مغزی شدید** | ⬜ |
| ⬜ | **سکته مغزی متوسط** | ⬜ |
| ⬜ | **نارسایی حاد کلیوی** | ⬜ |
| ⬜ | **آسیب کبدی** | ⬜ |

| سری 13 (از میان مشکلات زیر، کدامیک اگر برای شما رخ دهد بیشتر نگران و کدامیک رخ دهد کمتر نگران می شوید؟) | | |
| --- | --- | --- |
| **بیشترین نگرانی**(تنها یک گزینه انتخاب شود) | مشکلات سلامت | **کمترین نگرانی**(تنها یک گزینه انتخاب شود) |
| ⬜ | **درد عضلانی** | ⬜ |
| ⬜ | **حمله قلبی شدید** | ⬜ |
| ⬜ | **اثرات جانبی** | ⬜ |
| ⬜ | **سکته مغزی متوسط** | ⬜ |

**بخش سوم: نظر سنجی سوالات پرسشنامه**

1. فهمیدن سوالات برای من آسان بود.

کاملا موافقم موافقم نه موافقم و نه مخالفم کاملا مخالفم مخالفم

1. پاسخ دادن به سوالات برای من آسان بود.

کاملا موافقم موافقم نه موافقم و نه مخالفم کاملا مخالفم مخالفم

1. تمامی سوالات را بر اساس ترجیحاتم پاسخ دادم.

کاملا موافقم موافقم نه موافقم و نه مخالفم کاملا مخالفم مخالفم

**ویژگی های فردی شرکت کنندگان:**

1. نام شهرستان: ...........
2. نوع مرکز بهداشتی: شهری⬜ روستایی⬜
3. نام مرکز بهداشتی:............
4. سال تولد: ........
5. جنسیت: زن ⬜ مرد ⬜
6. وضعیت تاهل در حال حاضر: مجرد ⬜ متاهل ⬜ جدا شده ⬜ بیوه ⬜
7. تحصیلات: بی‌سواد ⬜ ابتدایی ⬜ راهنمایی ⬜ دبیرستان ⬜ دیپلم ⬜ دانشگاهی ⬜ حوزوی ⬜
8. محل سکونت: شهر ⬜ روستا ⬜
9. نحوه سکونت: به تنهایی ⬜ با اعضای خانواده⬜ با دیگران⬜
10. شغل: کارمند ⬜ کارگر ⬜ آزاد ⬜ بازنشسته ⬜ محصل ⬜ بیکار ⬜ خانه دار ⬜ سایر …..
11. سابقه مصرف استاتین (در گذشته یا حال حاضر): خیر ⬜ بلی درحال حاضر ⬜ بلی درگذشته ⬜
12. در صورت بلی، آیا تاا به حال دچار عارضه یا عوارض بعلت مصرف استاتین شده اید؟ بلی ⬜ خیر ⬜
13. نام عارضه مصرف استاتین را بیان نمایید. ........................
14. سابقه ابتلا به بیماری های مزمن (فشار خون بالا، چربی خون بالا، دیابت نوع 2، سرطان، بیماری روانی و ... ): خیر ⬜ بلی ⬜ (در صورت بلی به سوالات 15تا 25 پاسخ دهید.)
15. فشار خون بالا: بلی ⬜ خیر ⬜
16. در صورت بلی آیا سابقه مصرف داروهای کاهنده پرفشاری خون بالا را دارید؟ بلی ⬜ خیر ⬜
17. چربی خون بالا: بلی ⬜ خیر ⬜
18. در صورت بلی آیا سابقه مصرف داروهای کاهنده چربی خون بالا را دارید؟ بلی ⬜ خیر ⬜
19. دیابت نوع2 بلی ⬜ خیر ⬜
20. در صورت بلی آیا سابقه مصرف داروهای کاهنده قند خون بالا را دارید؟ بلی ⬜ خیر ⬜
21. سرطان: بلی ⬜ خیر ⬜
22. در صورت بلی آیا سابقه مصرف داروهای ضد سرطان را دارید؟ بلی ⬜ خیر ⬜
23. بیماری های روانی (افسردگی، اضطراب، وسواس، اختلال دوقطبی و اسکیزوفرنیا و... ): بلی ⬜ خیر ⬜
24. در صورت بلی آیا سابقه مصرف داروهای روانپزشکی را دارید؟ بلی ⬜ خیر ⬜
25. سایر بیماری ها را نام ببرید........
26. آیا خود شما، سابقه ابتلا به بیماری قلبی عروقی مانند سکته قلبی و یا سکته مغزی را دارید؟ بلی ⬜ خیر ⬜ (در صورت بلی به سوالات 27 تا 30 پاسخ داده شود)
27. سابقه سکته قلبی: بلی ⬜ خیر ⬜
28. چند سال است که سابقه سکته قلبی دارید؟ ....... سال
29. سابقه سکته مغزی: بلی ⬜ خیر ⬜
30. چند سال است که سابقه سکته مغزی دارید؟ ..... سال
31. آیا در بستگان درجه یک شما (پدر، مادر، خواهر، برادر) سابقه سکته قلبی، سابقه بستری در سی سی یو، سابقه جراحی قلب باز، سابقه آنژیوپلاستی، سابقه آنژیوگرافی مثبت، سابقه اسکن قلب مثبت، سابقه فوت بدلیل سکته قلبی وجود دارد؟ بلی ⬜ خیر ⬜ نمیدانم ⬜
32. آیا در بستگان درجه یک شما (پدر، مادر، خواهر، برادر) سابقه سکته مغزی و یا سابقه فوت بدلیل سکته مغزی وجود دارد؟ بلی ⬜ خیر ⬜ نمیدانم ⬜
33. آیا در بستگان درجه یک شما (پدر، مادر، خواهر، برادر) سابقه پرفشاری خون بالا وجود دارد؟بلی ⬜ خیر ⬜ نمیدانم ⬜
34. آیا در بستگان درجه یک شما (پدر، مادر، خواهر، برادر) سابقه چربی خون بالا وجود دارد؟بلی ⬜خیر ⬜ نمیدانم ⬜
35. آیا در بستگان درجه یک شما (پدر، مادر، خواهر، برادر) سابقه قند خون بالا وجود دارد؟بلی⬜ خیر ⬜ نمیدانم ⬜
36. بنظر شما مهمترین عامل تاثیر گذار بر اولویت بندی شما درخصوص مشکلات سلامت کدام می باشد؟

در دسترس بودن مراقبت های پزشکی ⬜

شدت مشکلات سلامت ⬜

علاج پذیری مشکلات سلامت ⬜

عواقب دارز مدت آن مشکل ⬜

مقرون بصرفه بودن خدمات پزشکی مرتبط ⬜

سایر دلایل (ذکر نمایید): .... .....
